# The association of modifiable and socio‐demographic factors with first transitions from smoking to exclusive e‐cigarette use, dual use or no nicotine use: Findings from the Avon Longitudinal Study of Parents and Children United Kingdom birth cohort

**DOI:** 10.1111/add.70076

**Published:** 2025-05-13

**Authors:** Alexandria Andrayas, Jon Heron, Jasmine Khouja, Hannah Jones, Marcus Munafò, Hannah Sallis, Lindsey Hines, Elinor Curnow

**Affiliations:** ^1^ School of Psychological Science University of Bristol Bristol UK; ^2^ MRC Integrative Epidemiology Unit University of Bristol Bristol UK; ^3^ Population Health Sciences, Bristol Medical School University of Bristol Bristol UK; ^4^ NIHR Bristol Biomedical Research Centre University Hospitals Bristol and Weston NHS Foundation Trust Bristol UK; ^5^ Centre for Academic Mental Health, Bristol Medical School University of Bristol Bristol UK; ^6^ Department of Psychology University of Bath Bath UK

**Keywords:** ALSPAC, e‐cigarettes, epidemiology, participant characteristics, risk factors, smoking cessation, young adults

## Abstract

**Background and Aims:**

E‐cigarettes can aid smoking cessation and reduce carcinogen exposure. Understanding differences in characteristics between young adults who quit smoking, with or without e‐cigarettes, or dual use can help tailor interventions. The aim of this study was to describe first transitions from smoking and explore substance use, sociodemographic, and health characteristic associations with the probability of each possible first transition from smoking.

**Design and Setting:**

Longitudinal birth cohort data from the Avon Longitudinal Study of Parents and Children (ALSPAC), conducted in the United Kingdom.

**Participants:**

A total of 858 participants were included who reported tobacco smoking in the past month at age 21 during a questionnaire collected in 2013.

**Measurements:**

The first reported non‐exclusive smoking event following smoking, observed approximately annually between ages 22 and 30, was categorized as either no nicotine use, exclusive e‐cigarette use, or dual use. Discrete‐time subdistribution hazard models were used to examine associations between different covariates, including substance use, sociodemographic, and health characteristics, with the probability of each first transition from smoking. Analyses were adjusted for early‐life confounders and weighted to mitigate bias.

**Findings:**

Among participants, 52% stopped nicotine use, 27% reported dual use, and 9% used e‐cigarettes exclusively. Smoking weekly or more (Subdistribution Hazard Ratio [SHR] = 0.28, 95% Confidence Interval [CI] = 0.22–0.35), having many friends who smoke (SHR = 0.64, 95% CI = 0.50–0.81), and lower education (SHR = 0.68, 95% CI = 0.52–0.90) reduced the likelihood of no nicotine use and increased dual use (*frequent smoking* SHR = 3.00, 95% CI = 1.96–4.59; *peer smoking* SHR = 1.55, 95% CI = 1.07–2.24; *education* SHR = 1.72, 95% CI = 1.03–2.90). Cannabis use (SHR = 0.67, 95% CI = 0.49–0.92), drug use (SHR = 0.77, 95% CI = 0.59–0.99), less exercise (SHR = 0.71, 95% CI = 0.53–0.95), and early parenthood (SHR = 0.46, 95% CI = 0.27–0.79) reduced no nicotine use. Higher BMI (SHR = 1.58, 95% CI = 1.08–2.31) increased dual use.

**Conclusions:**

In the United Kingdom, young adults who smoke frequently, have more smoking peers, have lower education, engage in drug use, exercise less, or become parents early appear to be less likely to stop nicotine use than other young adults who smoke. Frequent smoking, peer smoking, lower education, and higher body mass index also appear to be associated with increased dual use of cigarettes and e‐cigarettes.

## INTRODUCTION

Tobacco control approaches in the United Kingdom (UK) have achieved great success given smoking rates have been declining since records began in 1974 [1]. However, harm reduction is essential for individuals who smoke tobacco and have either struggled to quit or do not intend to. One potential harm reduction tool is electronic cigarettes (e‐cigarettes), which may support smoking cessation. E‐cigarettes are devices that heat liquid, often containing propylene glycol, vegetable glycerine, flavourings, nicotine and other additives, to form an aerosol that can be inhaled. The aerosol contains lower levels of selected toxicants compared to cigarette smoke [2]. When used for smoking cessation, nicotine e‐cigarettes can be more successful than traditional nicotine replacement therapy (NRT), nicotine‐free e‐cigarettes, or behavioural support alone [3, 4].

Since emerging on the UK market in 2007 [5], e‐cigarettes have grown in popularity. An estimated 4.7 million adults in Great Britain used e‐cigarettes in 2023, 56% of whom previously smoked, and 37% of whom currently smoke [6]. Recent reduction in smoking rates may be partly driven by the availability of e‐cigarettes, which may attract people who would not have otherwise used NRT [7, 8, 9, 10]. E‐cigarettes have been regulated as consumer products as part of the UK Tobacco and Related Products Regulations since 2016 [11, 12, 13]. Recently the UK government and National Health Service pioneered a Swap to Stop campaign aimed to help people quit smoking by offering free vape starter kits and behavioural support [14]. However, not all people who smoke switch to solely vaping or use e‐cigarettes to try to stop smoking.

When people continue to smoke alongside using e‐cigarettes this is referred to as dual use. Dual use is associated with greater nicotine dependence and consumption of both cigarettes and e‐cigarettes [15], may expose a person to similar levels of carcinogens as only smoking [16], and is unlikely to substantially reduce harm if it does not lead to quitting smoking [17]. Therefore, it is important to understand the factors associated with dual use to target those most in need of support to effectively use e‐cigarettes to stop smoking.

Previous research has identified predictors of e‐cigarette use, including higher tobacco dependence [18], smoking by friends or family and higher impulsivity [19], internalising mental health symptoms [20], drunkenness, energy drink use and poor academic achievement [21] and conduct problems [22]. Studies have also shown that e‐cigarette use may be less persistent over time than cigarette use [23, 24, 25] and may facilitate a quicker transition to smoking abstinence [26]. However, much of this research has focused on adolescence or the entire adult age range, has limited follow‐up data up to a few years and has focused on one or few predictors. This study adds to existing knowledge by using 8 years of multiple follow ups in a rich longitudinal dataset of young adults in the United Kingdom.

This study investigates differences in a broad range of characteristics between young adults in the United Kingdom who previously reported smoking and subsequently reported either using e‐cigarettes exclusively or while smoking (dual use) or quit smoking without using e‐cigarettes. Understanding the factors associated with different initial transitions from smoking can help identify target populations who are less likely to quit smoking or use e‐cigarettes, or more likely to dual use.

### Aims

This study aims to (1) report the first self‐reported changes in nicotine use, including transitions to no nicotine use, exclusive e‐cigarette use and dual use in participants who previously reported smoking in the Avon Longitudinal Study of Parents and Children (ALSPAC); and (2) explore the relative strength of associations between substance use, socio‐demographic and health characteristics with the probability of each possible first transition from smoking.

## METHODS

### Participants

Pregnant women resident in Avon, United Kingdom with expected delivery dates between 1 April 1991 and 31 December 1992 were invited to take part in the study. Briefly, 14 541 women were initially recruited, resulting in 14 062 live births and 13 988 children alive at 1 year, and additional children were subsequently enrolled. The study has been described at length previously [27, 28, 29, 30, 31]. Further details related to the ALSPAC sample can be found in Text S1.

The eligible sample for the present analyses (*n* = 3290) consists of G1 participants in ALSPAC who responded to the 21+ questionnaire (administered from mid‐December 2013 when participants were ~21 years of age) and reported whether they had smoked in the past 30 days. The analytic sample (*n* = 858) is limited to participants who responded ‘yes’ to smoking in the past 30 days. Of these 803 (94%) participants also reported their smoking and e‐cigarette use behaviour at least once in a following timepoint. Attrition and selection into the sample is illustrated in Figure S1.

### Design

This is a secondary analysis of ALSPAC, a longitudinal birth cohort survey. Data used in the present study were collected via self‐reported questionnaires and clinic attendance. This analysis focuses on participants who had smoked in the past month at 21 years of age and subsequent nicotine use from 22 to 30 years of age.

### Outcomes

Nicotine use status at each timepoint, where smoking and e‐cigarette use data have been collected, (22, 23, 24, 28 and 30 years) was categorised as either ‘exclusive smoking’ (smoked in the past month but not currently using e‐cigarettes), ‘no nicotine use’ (no smoking or e‐cigarette use), ‘exclusive e‐cigarette use’ (no smoking but currently using e‐cigarettes) or ‘dual use’ (smoking and e‐cigarette use). To note, we use the term ‘no nicotine use’ for conciseness, but this definition refers to the use of nicotine products assessed in this study (i.e. cigarettes and e‐cigarettes) and does not consider other sources of nicotine.

The first transition following smoking at 21 years, if any, was determined based on the first reported non‐exclusive smoking event (nicotine use status) during the 22‐ to 30‐year time period. Participants' first‐reported transitions from smoking were then derived by identifying the earliest follow‐up (~1, 2, 3, 7 or 9 years after the 21+ questionnaire) at which either no nicotine use, exclusive e‐cigarette use or dual use was reported. Those who only reported exclusive smoking by the final follow‐up were assigned the maximum time. An illustrative example is shown in Figure S2.

### Exposures

Exposures of interest were determined through discussion among the study team and examination of the literature. We have summarised relevant existing literature in Table S1. Eleven dichotomised substance use, socio‐demographic and health characteristics were investigated, spanning a wide range of potential intervention targets. Available measures collected during or in the 3 years before the 21+ questionnaire were selected.

#### Substance use

Measures related to substance use include smoking frequency [smoked in past month but less than weekly (reference category) vs. weekly or more] at age 21 years, and frequency of binge drinking six or more units of alcohol [never, monthly or less (ref) vs. weekly or more], past year cannabis use frequency [never, not in the past year, less than monthly (ref) vs. monthly or more] and past year drug use [no (ref) vs. yes], all collected at age 20 years.

#### Social and socio‐demographic factors

Measures related to social and socio‐demographic factors include peer smoking, referring to the number of friends who had smoked cigarettes from 18 years of age until now [none, a few or some (ref) vs. most or all], educational attainment [degree‐level (ref) vs. A‐level, below or other], where A‐level (‘Advanced Level’) qualifications typically follow General Certificate of Secondary Education (GCSE) examinations or equivalent, usually in the age range of 16 to 18 years, both collected at age 20 years, and early parenthood [no (ref) vs. yes] and neighbourhood deprivation, estimated via Townsend deprivation scores [least deprived quintile (ref) vs. more deprived quintiles], both collected at age 21 years.

#### Physical and mental health

Measures related to physical and mental health include body mass index (BMI) [less than 25 (ref) vs. 25 or more] and frequency of exercise in the past year [weekly or more (ref) vs. less than weekly], both collected at age 18 years, and depressive symptoms in the previous 2 weeks, estimated via Short Mood and Feelings Questionnaire (SMFQ) scores [less than 12 (ref) vs. 12 or more], collected at age 21 years. Higher scores on the SMFQ suggest more severe depressive symptoms, where a score of 12 or higher may indicate the presence of depression.

### Confounders

Analyses were adjusted for four early‐life confounders; sex assigned at birth [male (ref) vs. female], ethnicity [White (ref) vs. minority ethnic], average household income (Great Britain Pound) per week at age 11 [560+ (ref) vs. 430–559, 240–429, <240] and parental smoking [no (ref) vs. yes] reported by the mother and/or her partner when the participant was age 12 years. Although it is best to use precise terminology to describe the specific ethnicity of a person or group, these data were unavailable. Instead, ALSPAC derived ethnicity based on the reported ethnic groups of the mother and father. Here, we use ‘minority ethnic’ to refer to participants from all other ethnic groups combined when compared to participants with two White parents. This uses ‘minority’ in a United Kingdom context, but these groups often represent the global majority.

### Statistical analysis

Data were cleaned and variables created using Stata (version 18) [32] removing participants who had withdrawn their consent by the time data were accessed. Statistical analyses and data visualisation were carried out in R (version 4.3.3) [33]. This study adhered to the STROBE guidelines for the reporting of observational studies (Table S2) [34].

Subdistribution hazard discrete‐time models [35, 36] were used to gain insight into covariate associations with the probability of each first‐reported transition from smoking. These models can accommodate competing risks in a discrete time setting where participants experiencing a competing non‐exclusive smoking event remain in the risk set given nicotine use status is transitory. These models were used to investigate 11 substance use, socio‐demographic and health characteristic (18–21 years) associations with the relative likelihood of each transition from smoking (22–30 years) over time. Results describe the relative change in the subdistribution hazard ratio associated with each characteristic adjusted for four baseline confounders. Further details related to the statistical analysis approach can be found in Text S2.

Multiple imputation by chained equations was used to impute any missing data [37, 38]. Details on missingness and how it was dealt with in this sample [39] can be found in Text S3, Figures S3–S7 and Tables S3–S4. As the analytic sample is conditioned on smoking status and this may induce bias, downstream discrete‐time survival analyses were weighted by the propensity of participants to report past‐month smoking at age 21 years [40, 41]. This is described in Text S4 and illustrated in Figure S8.

There were limited data on subsequent transitions (i.e. after the first transition from smoking) hence the focus of the present study is on the first reported non‐exclusive smoking event. However, all observed transitions are described in Text S5 and shown in Tables S5–S6.

## RESULTS

### Descriptive statistics

Reported exclusive smoking declined with time, while no nicotine use and e‐cigarette use increased with time. Initially dual use was more commonly reported than exclusive e‐cigarette use, however, at later timepoints similar proportions are observed (Figure S9).

In the analytic sample (*n* = 858), only 8% of participants reported being a parent at 21 years and only 4% had at least one parent from a minority ethnic background (Table 1). Frequency of exercise measured in the 18‐year questionnaire showed the most missingness (44%) out of the investigated characteristics. Differences in participant characteristics by whether or not any transition was reported, and by each first transition from smoking can be found in Tables S7–S8. Table S9 shows differences in participant characteristics by the four included early‐life confounders.

**TABLE 1:** Participant characteristics (*n* = 858).

Characteristic	Missing *n* (%)	Non‐missing *n* (%)
Early‐life confounders		
Sex assigned at birth	0 (0)	
Male		308 (36)
Female		550 (64)
Ethnicity	70 (8.2)	
White		760 (96)
Minority ethnic		28 (3.6)
Household income (11 y)	207 (24)	
560+		265 (41)
430–559		141 (22)
240–429		176 (27)
<240		69 (11)
Parental smoking (12 y)	137 (16)	
No		547 (76)
Yes		174 (24)
Substance use		
Smoking frequency (21 y)	5 (0.6)	
Occasional		312 (37)
Weekly or more		541 (63)
Binge drinking frequency (20 y)	228 (27)	
Never, monthly or less		353 (56)
Weekly or more		277 (44)
Cannabis use (20 y)	215 (25)	
Never, less than monthly or not in past year		489 (76)
Monthly or more		154 (24)
Drug use (20 y)	245 (29)	
No		347 (57)
Yes		266 (43)
Social and socio‐demographic factors		
Peer smoking (20 y)	218 (25)	
None, a few or some		261 (41)
Most or all		379 (59)
Education (20 y)	211 (25)	
Degree‐level		134 (21)
A‐level or lower/other		513 (79)
Being a parent (21 y)	9 (1.0)	
No		779 (92)
Yes		70 (8.2)
Neighbourhood deprivation (21 y)	23 (2.7)	
Least deprived quintile		293 (35)
More deprived quintiles		542 (65)
Physical and mental health		
BMI (18 y)	253 (29)	
<25		470 (78)
≥25		135 (22)
Exercise frequency (18 y)	375 (44)	
Weekly or more		314 (65)
Less than weekly		169 (35)
Depressive symptoms (21 y)	30 (3.5)	
<12 SMFQ score		653 (79)
≥12 SFMQ score		175 (21)

Abbreviations: BMI, body mass index; SMFQ, Short Mood and Feelings Questionnaire (%); y, approximate years of age.

### First self‐reported changes in nicotine use

**FIGURE 1:**
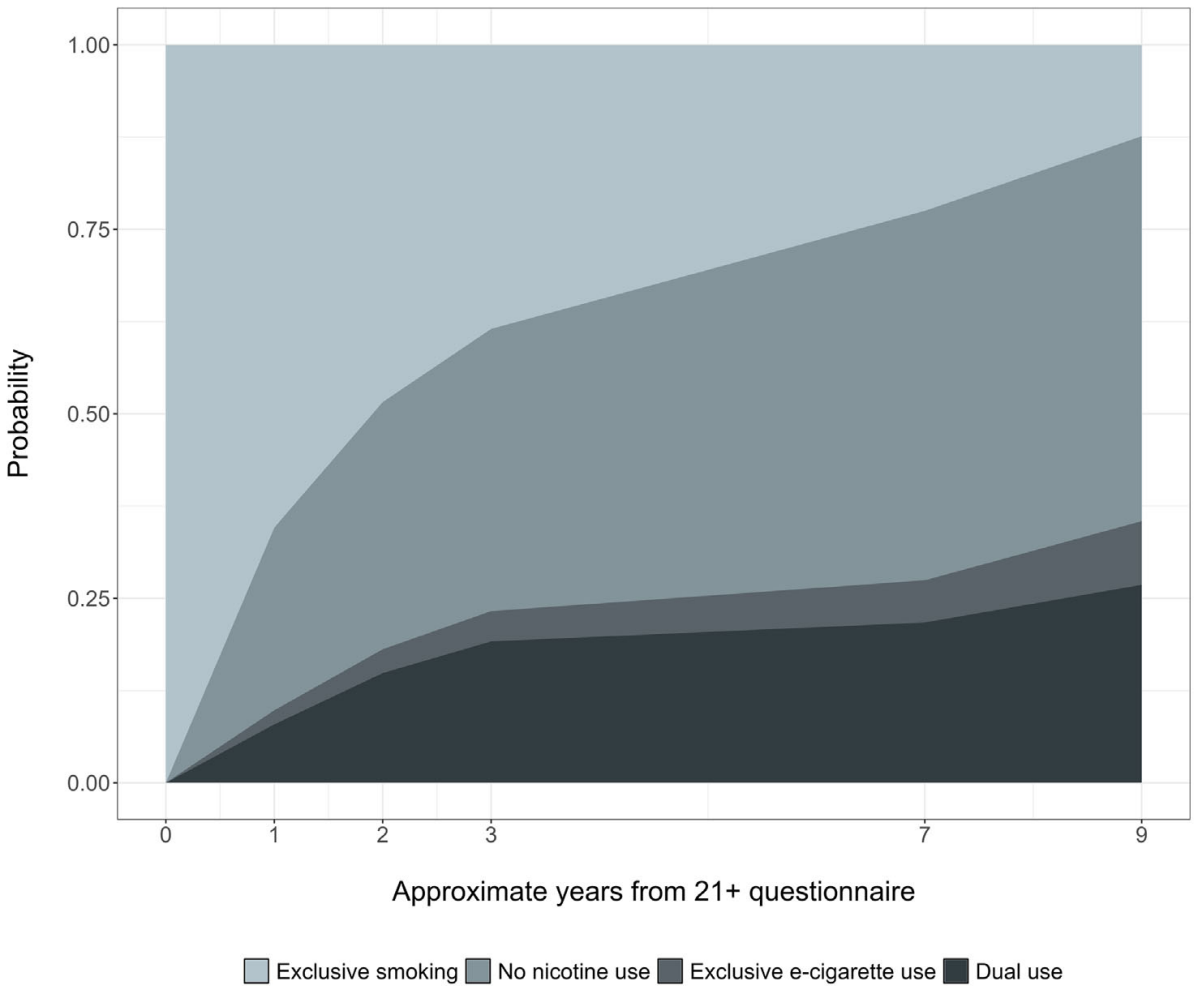
Stacked cumulative incidence curves showing the probability of each first transition from smoking over time since 21+ questionnaire.

By the end of follow‐up 12% of participants were still exclusively smoking and had not reported quitting or using e‐cigarettes at any point (Figure 1). Of those with an observed non‐exclusive smoking event, most first reported no nicotine use (52%) and a large proportion of initial transitions to no nicotine use occurred approximately 1 year following the 21+ questionnaire. Transitions from smoking to first using e‐cigarettes were seen in 36% of participants and more participants reported dual use (27%) than exclusive e‐cigarette use (9%).

### Associations between characteristics with probability of each transition

**FIGURE 2:**
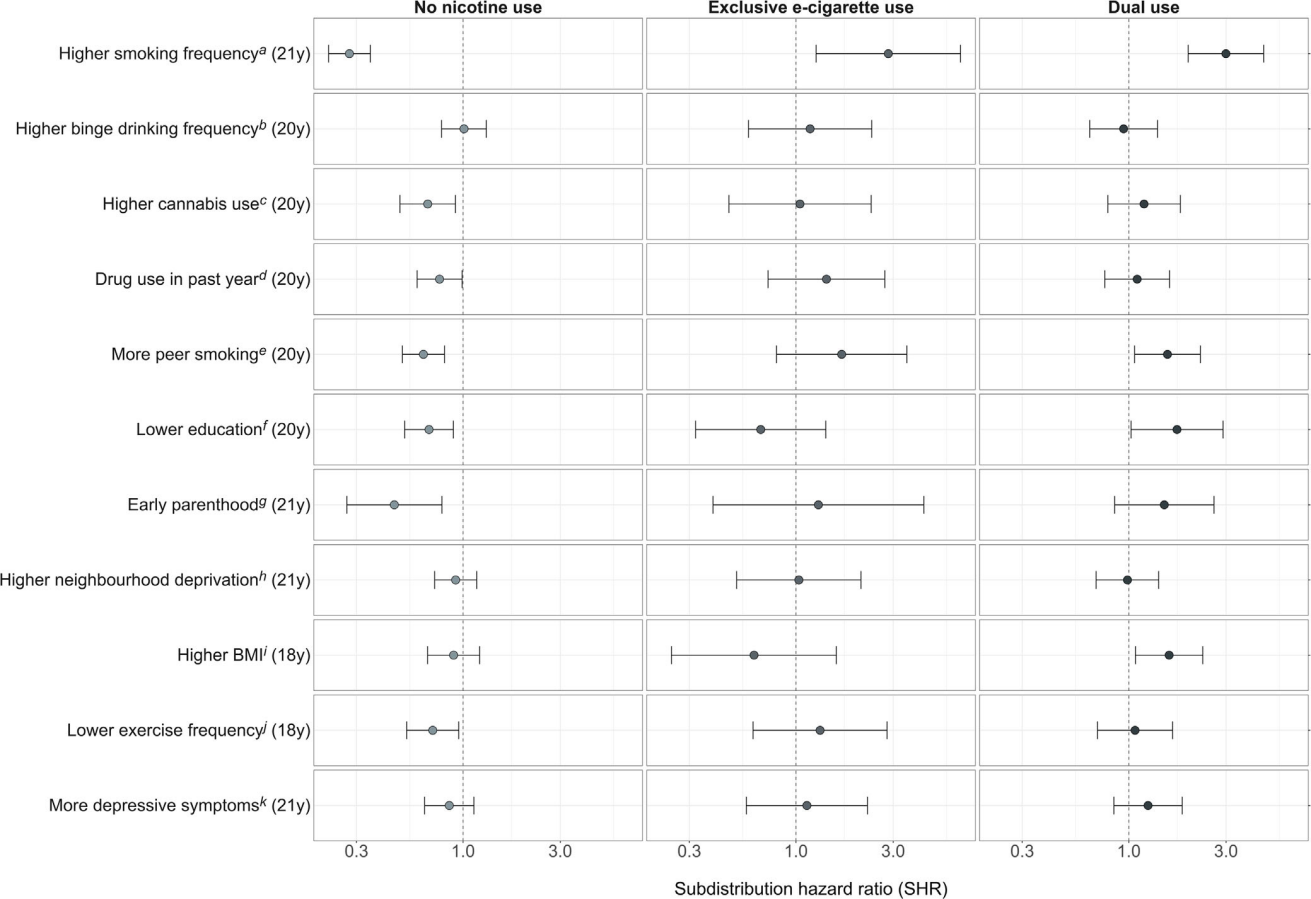
Pooled results from subdistribution discrete‐time survival analyses, adjusted for sex, ethnicity, household income at 11 years (y) and parental smoking (12 y) and weighted for selection via smoking. ^a^Smoked in past month but less than weekly (ref) versus weekly or more; ^b^never drinks more than 6 units, drinks monthly, or less (ref) versus weekly or more; ^c^never used cannabis, not in the past year, less than monthly (ref) versus monthly or more; ^d^never used drugs or not in past year (ref) versus used in past year; ^e^no friends smoke, a few, or some (ref) versus most or all; ^f^degree‐level (ref) versus A‐level, below, or other qualification; ^g^not a biological or non‐biological parent (ref) versus is a parent; ^h^least deprived Townsend deprivation score quintile (ref) versus more deprived quintiles; ^i^body mass index (BMI) less than 25 (ref) versus 25 or more; ^j^exercised weekly or more in past year (ref) versus less than weekly; ^k^short mood and feelings questionnaire (SMFQ) score of less than 12 (ref) versus score of 12 or more.

Figure 2 and Table 2 show the pooled results related to the 11 main characteristics of interest after adjustment for early‐life confounders and weighting for selection via smoking. These are discussed below.

**TABLE 2:** Pooled summary statistics from subdistribution discrete‐time survival analyses, adjusted for early‐life confounders, weighted for selection via smoking.

Characteristic	No nicotine use (52%)			Exclusive e‐cig use (9%)			Dual use (27%)		
	SHR	95% CI	P	SHR	95% CI	P	SHR	95% CI	P
Substance use									
Smoking frequency (21 y)									
Occasional	–	–	–	–	–		–	–	
Weekly or more	0.28	0.22–0.35	<0.001	2.84	1.26–6.42	0.012	3.00	1.96–4.59	<0.001
Binge drinking frequency (20 y)									
Never, monthly or less	–	–	–	–	–	–	–	–	
Weekly or more	1.01	0.78–1.30	>0.9	1.17	0.58–2.36	0.700	0.94	0.64–1.38	0.800
Cannabis use (20 y)									
Never, less than monthly, not past year	–	–	–	–	–	–	–	–	–
Monthly or more	0.67	0.49–0.92	0.013	1.05	0.47–2.34	>0.9	1.19	0.79–1.79	0.400
Drug use (20 y)									
No	–	–	–	–	–	–	–	–	–
Yes	0.77	0.59–0.99	0.042	1.41	0.73–2.73	0.300	1.10	0.76–1.58	0.600
Social and socio‐demographic factors									
Peer smoking (20 y)									
None, a few, or some	–	–	–	–	–	–	–	–	–
Most or all	0.64	0.50–0.81	<0.001	1.68	0.80–3.50	0.200	1.55	1.07–2.24	0.021
Education (20 y)									
Degree‐level	–	–	–	–	–	–	–	–	–
A‐level or lower/other	0.68	0.52–0.90	0.006	0.67	0.32–1.40	0.300	1.72	1.03–2.90	0.040
Being a parent (21 y)									
No	–	–	–	–	–	–	–	–	–
Yes	0.46	0.27–0.79	0.005	1.29	0.39–4.24	0.700	1.49	0.85–2.62	0.200
Neighbourhood deprivation (21 y)									
Least deprived quintile	–	–	–	–	–	–	–	–	–
More deprived quintiles	0.92	0.73–1.17	0.500	1.03	0.51–2.08	>0.9	0.98	0.69–1.40	>0.9
Physical and mental health									
BMI (18 y)									
<25	–	–	–	–	–	–	–	–	–
≥25	0.90	0.67–1.21	0.500	0.62	0.25–1.58	0.300	1.58	1.08–2.31	0.019
Exercise frequency (18 y)									
Weekly or more	–	–	–	–	–	–	–	–	–
Less than weekly	0.71	0.53–0.95	0.023	1.31	0.62–2.80	0.500	1.07	0.70–1.64	0.700
Depressive symptoms (21 y)									
<12 SMFQ score	–	–	–	–	–	–	–	–	–
≥12 SMFQ score	0.86	0.65–1.13	0.300	1.13	0.57–2.24	0.700	1.24	0.85–1.83	0.300

Abbreviations: e‐cig, electronic cigarette; SHR, subdistribution hazard ratio, SMFQ, Short Mood and Feelings Questionnaire.

#### Substance use

Smoking frequency at age 21 years was the only characteristic associated with the probability of all possible first transitions from smoking. Participants who smoked weekly or more were less likely to transition to no nicotine use [subdistribution hazard ratio (SHR) = 0.28, 95% CI = 0.22–0.35], and more likely to first report exclusive e‐cigarette use (SHR = 2.84, 95% CI = 1.26–6.42) and dual use (SHR = 3.00, 95% CI = 1.96–4.59) as opposed to a competing event or no event, at any given time, compared to participants who smoked less frequently.

There was no evidence to suggest binge drinking more regularly was associated with the probability of any transitions from smoking in this study. Participants who, at age 20 years, reported using cannabis monthly or more in the past year (SHR = 0.67, 95% CI = 0.49–0.92), or using other drugs in the past year (SHR = 0.77, 95% CI = 0.59–0.99) were less likely to transition to no nicotine use, as opposed to using e‐cigarettes or continuing exclusive smoking, compared to those who did not use drugs. There was little to no evidence that other substance use was associated with the probability of first using e‐cigarettes.

#### Social and socio‐demographic factors

Participants who reported that most or all their friends smoked were less likely to transition to no nicotine use (SHR = 0.64, 95% CI = 0.50–0.81), and more likely to first transition to dual use (SHR = 1.55, 95% CI = 1.07–2.24) compared to participants with fewer friends who smoked.

Participants who reported having fewer educational qualifications at age 20 years were also less likely to first transition to no nicotine use (SHR = 0.68, 95% CI = 0.52–0.90) and more likely to first report dual use (SHR = 1.72, 95% CI = 1.03–2.90) following smoking, compared to those with higher education.

There was weak evidence to suggest peer smoking and education was associated with the probability of first transitions to exclusive e‐cigarette use given the large amount of uncertainty reflected in the wide CI. However, the point estimate related to the association of peer smoking with exclusive e‐cigarette use was similar as for dual use. Conversely the point estimate reflecting the association of education with exclusive e‐cigarette use showed the opposite direction of association compared to dual use.

Participants who reported being a parent at age 21 years were less likely to first transition to no nicotine use (SHR = 0.46, 95% CI = 0.27–0.79) compared to non‐parents. There was little to no evidence that parenthood was associated with the probability of first using e‐cigarettes.

There was no clear evidence to suggest neighbourhood deprivation at age 21 years was associated with the probability of any first transitions from smoking in this study.

#### Physical and mental health

Participants with a BMI over or equal to 25 at age 18 years were more likely to first transition to dual use (SHR = 1.58, 95% CI = 1.08–2.31) than those with a lower BMI. There was little to no evidence that BMI was associated with the probability of first transitions to no nicotine use, or to exclusive e‐cigarette use.

Participants who exercised less than weekly in the past year at age 18 were less likely to transition to no nicotine use (SHR = 0.71, 95% CI = 0.53–0.95) compared to more active participants. There was little to no evidence that exercise frequency was associated with the probability of transitions to using e‐cigarettes following smoking.

There was little to no evidence to suggest that higher depressive symptoms at age 21 years was associated with the probability of any first transitions from smoking in this study.

#### Other analyses

Results related to the four early‐life confounders are shown in Table S10, but not discussed here as these coefficients cannot be interpreted as the effect of each on transitions from smoking because of mutual adjustment.

Results from unweighted, unadjusted and complete case analyses are shown in Tables S11–S13. Results from unweighted and unadjusted analyses were similar to those described above, while the unweighted complete case analyses showed some differences and are described in Text S3.

## DISCUSSION

In a sample of young adults who smoked tobacco, this study explored predictors (18–21 years) of first reported transitions from smoking (21 years) to no nicotine use, exclusive e‐cigarette use and dual use, in early adulthood (22–30 years) within a UK setting.

There were many barriers to stopping nicotine use identified, where complex lifestyle behaviours and stressors may make quitting tobacco use more difficult. Despite the increased prevalence of e‐cigarette use amongst those who previously or currently smoked [1], some groups may not take up e‐cigarettes such as people who exercise less, use drugs or became parents early. The continued use of tobacco in these groups is likely to exacerbate existing health inequalities as those using cannabis are at increased risk of mental health disorders [42, 43], the combined use of tobacco and lower physical activity has additive impacts on poor physical health [44], and continued use of tobacco amongst parents makes next generations more likely to smoke themselves [45]. Consequently, these groups represent key targets for smoking cessation and harm reduction interventions.

Several groups who were less likely to report no nicotine use were more likely to first transition to dual use. These include people who smoke more, have lower educational attainment, or more friends who smoke. Findings are mixed regarding harm reduction via dual use. There is evidence that dual use is related to reduced tobacco cigarette consumption [46], but other evidence indicates that dual use may not provide substantial harm reduction [16, 17, 47]. It is unknown whether these individuals used e‐cigarettes with an aim of ceasing smoking, as previously reported motivations for dual use focus on pleasure rather than desire to quit [15]. Dual use may then reflect ambivalence toward quitting or an attempt to mitigate harm while maintaining nicotine dependence. Nevertheless, our findings suggest that dual use may be particularly common among people with more entrenched smoking habits or social influences, who may in turn not feel ready or able to fully quit smoking. Understanding how these groups can be encouraged to solely use e‐cigarettes, which has lower impacts on health than dual use [47], is then an important step for public health. Our findings suggest that while smoking frequency and peer smoking may influence e‐cigarette use generally, education and BMI may distinguish whether individuals exclusively use e‐cigarettes or dual use. This supports previous work showing how e‐cigarette use often co‐exists with tobacco use within lower socio‐economic groups rather than displacing it [48, 49].

Current research into e‐cigarette use often focusses on individuals who previously did not smoke [50, 51], whereas our focus on individuals who already used tobacco fulfils important gaps in the literature and offers insights and implications for tobacco cessation and harm reduction. Current intervention to increase tobacco cessation focusses on a variety of strategies such as behavioural therapies [52], pharmacological treatments [53], financial incentives [54] and innovative digital approaches [55, 56, 57, 58]. Tailored programmes that help people who smoke switch to less harmful alternatives to smoking and help people who dual use switch completely to e‐cigarettes, could be developed and better promoted toward people who are more demographically vulnerable or dependant on cigarettes [59]. However, currently the effects of tailoring smoking cessation interventions for disadvantaged people have been limited [60], and programs that aim to integrate and address substance use, physical inactivity and smoking concurrently need to be improved [61]. Given e‐cigarettes are providing a new avenue to help people quit smoking, where behavioural support combined with e‐cigarette use has been shown to be particularly effective [62], it is important to ensure any ensuing reductions in smoking‐related harms are equitable [63].

### Strengths and limitations

The strengths of this study include the length of follow‐up data spanning young adulthood, the use of a phenotypically rich cohort with many measures of relevant risk factors, the use of a competing risk framework, and the use of multiple imputation and weights to mitigate complete case and selection bias, respectively, to garner more robust findings.

Limitations include the small sample size, measurement error where complex characteristics have been simplified and potential confounding or interactions between the characteristics being separately investigated. Left and interval censoring is another issue that may lead to less precise estimates. Current e‐cigarette use before 22 years was not known nor the exact timings of reported first transitions from smoking. People who occasionally smoke might also exhibit fluctuations that do not necessarily indicate sustained transitions. Other sources of nicotine such as NRT were not investigated. Findings may also be subject to attrition bias as continued participation in ALSPAC has been shown to be non‐random [64, 65].

The landscape of e‐cigarettes in the United Kingdom is rapidly evolving and increasing in popularity [66] where the use of e‐cigarettes within England among smokers and recent ex‐smokers plateaued from 2013 to 2020, but has grown since [67]. This makes it difficult to understand the influence of participant characteristics independent of their current technological, social and political contexts.

Larger and better powered studies will be needed to investigate how characteristics such as depressive symptoms or neighbourhood deprivation, which may contribute smaller effects and vary over time, influence transitions away from exclusive smoking. Such studies could also be used to explore subsequent transitions between nicotine use states (e.g. no nicotine use after using e‐cigarettes) via multi‐state model analysis. Future work should also examine the complex interplay between different characteristics and motivations on quitting smoking or using e‐cigarettes.

### Implications

We identified many groups who may be less motivated or able to quit smoking, and likely need greater efforts from stop smoking interventions. Some groups, such as people with higher BMI, lower education or more peers who smoke, do appear to be using e‐cigarettes, but often by combining e‐cigarette use with tobacco and, therefore, may not fully benefit from their harm reduction potential. Other groups such as people who are less active or use other substances may not be engaging with e‐cigarettes, or find it more difficult than others to feel encouraged to switch from smoking to vaping. Future policies and interventions should address the potential harms of dual use versus completely switching to e‐cigarettes and encourage safer alternative sources of nicotine, especially if they are unable or unwilling to quit smoking unaided or with other nicotine replacement methods.

## AUTHOR CONTRIBUTIONS

**Alexandria Andrayas**: Conceptualization (lead); formal analysis (lead); visualization (lead); writing—original draft preparation (lead); writing—review and editing (lead). **Jon Heron**: Conceptualization (equal); formal analysis (equal); supervision (equal); writing—review and editing (equal). **Jasmine Khouja**: Conceptualization (equal); formal analysis (equal); writing—review and editing (equal). **Hannah Jones**: Conceptualization (equal); formal analysis (equal); writing—review and editing (equal). **Hannah Sallis**: Funding acquisition (lead); conceptualization (lead); supervision (equal); writing—review and editing (equal). **Marcus Munafò**: Funding acquisition (equal); writing—review and editing (equal). **Lindsey Hines**: Conceptualization (equal); formal analysis (equal); supervision (lead); writing—review and editing (equal). **Elinor Curnow**: Conceptualization (equal); formal analysis (lead); visualization (lead); supervision (equal); writing—review and editing (equal).

## DECLARATION OF INTERESTS

None.

## Supporting information


**Text S1.** Additional information regarding sample.
**Text S2.** Additional information regarding statistical analysis.
**Text S3.** Missingness.
**Text S4.** Description of approach used to address potential bias induced by conditioning sample on smoking.
**Text S5.** Description of all observed transitions between nicotine use states.
**Text S6.** Divergence from pre‐registration.
**Figure S1.** Flowchart showing attrition and available sample size.
**Figure S2.** Illustrative example showing how first reported transitions from smoking are identified using synthetic data for five participants.
**Figure S3.** Causal diagram illustrating the relation of the analysis and auxiliary variables with missingness.
**Figure S4.** Correlation matrix showing relationships between variables used in imputation within the eligible sample of participants who responded at the 21 + questionnaire (n = 3,290) used to derive weights related to selection via smoking.
**Figure S5.** Correlation matrix showing relationships between variables used in imputation within the analytic sample of people who smoked in the past 30 days at the 21 + questionnaire (n = 858) used to investigate associations between participant characteristics and transitions from smoking using discrete time subdistribution hazard models.
**Figure S6.** Predictor matrix showing variables included in the imputation model within the eligible sample of participants who responded to the 21 + questionnaire (n = 3,290). All variables used in the selection models were included in each imputation model to ensure consistency in deriving weights related to selection via smoking.
**Figure S7.** Predictor matrix showing variables included in the imputation model within the analytic sample of participants who smoked in the past 30 days at the 21 + questionnaire (n = 858). All variables used in the analysis models were included in each imputation model to ensure consistency in investigating associations between participant characteristics and transitions from smoking using discrete‐time subdistribution hazard models.
**Figure S8.** Possible collider‐conditioning bias when investigating e‐cigarette use after conditioning on smoking and approach used to mitigate this bias.
**Figure S9.** Number of participants by self‐reported nicotine use and missingness at each timepoint in the analytic sample (n = 858).
**Table S1.** Background literature related to the investigated risk factors in the context of smoking tobacco and/or vaping e‐cigarettes.
**Table S2.** Checklist of items that should be included in reports of cohort studies.
**Table S3.** Participant characteristics in analytic sample with complete records for baseline confounders and investigated risk factors.
**Table S4.** Predictors (including all analysis variables and those used in imputation) of having a complete record.
**Table S5.** All observed transitions between nicotine use states across 5 timepoints, from ages 21 to 30 years, where nicotine use status when missing was assumed to remain the same as that reported during the previous wave of data collection. The numbers shown in brackets refer to the first reported transition observed following smoking at age 21.
**Table S6.** Average of all observed transitions between nicotine use states across 5 timepoints, from ages 21 to 30 years, where nicotine use status at all waves of data collection was imputed. The numbers shown in brackets refer to the first reported transition observed following smoking at age 21.
**Table S7.** Differences in characteristics between participants who did or did not report at least one transition between nicotine use states.
**Table S8.** Differences in characteristics by first reported transitions from smoking in those not loss to follow up, where transitions were derived by assuming transitions did not occur during any missing prior or intermediate reports of nicotine use, excluding any missingness in each characteristic.
**Table S9.** Differences in characteristics by early‐life confounders excluding any pairwise missingness.
**Table S10.** Pooled summary statistics for *early‐life confounders* and time from sub‐distribution discrete time survival analyses, weighted for selection via smoking.
**Table S11.** Pooled summary statistics from sub‐distribution discrete time survival analyses, adjusted for early‐life confounders, *unweighted* .
**Table S12.** Pooled summary statistics from sub‐distribution discrete time survival analyses, *unadjusted* , weighted for selection via smoking.
**Table S13.** Summary statistics from sub‐distribution discrete time survival, adjusted for early‐life confounders, *complete cases* (n = 168), unweighted (as weights rely on imputed data).

## Data Availability

Data used in this project and any resulting data from the analyses are available on request to the ALSPAC Executive Committee (alspac-exec@bristol.ac.uk) and subject to a data access fee. Study data were collected and managed using REDCap electronic data capture tools hosted at the University of Bristol [68]. The study website contains details of available data through a fully searchable data dictionary and variable search tool: http://www.bristol.ac.uk/alspac/researchers/our-data/. Ethical approval for the study was obtained from the ALSPAC Law and Ethics Committee and Local Research Ethics Committees (NHS Haydock REC: 10/H1010/70). Informed consent for the use of data collected via questionnaires and clinics was obtained from participants following the recommendations of the ALSPAC Ethics and Law Committee at the time. Consent for biological samples has been collected in accordance with the Human Tissue Act (2004). Data access for this project was granted (B3499/B4347) before this study. Datasets were created using syntax templates from the 25 January 2024. The proposed analysis of data was pre‐registered on the Open Science Framework (OSF) here: https://osf.io/nuz5b. Deviations from this pre‐registration are described in Text S6. The code used for data analysis is available in a github repository here: https://github.com/alexandrayas/ALSPAC_CRUK_smkvap/tree/main/Transitions%20from%20smoking.
