# Supplementary material for: The association of modifiable and socio‐demographic factors with first transitions from smoking to exclusive e‐cigarette use, dual use or no nicotine use: Findings from the Avon Longitudinal Study of Parents and Children United Kingdom birth cohort
Source: Addiction. 2025 May 13;120(9):1804–15. doi: 10.1111/add.70076 (PMC12319662; doi:10.1111/add.70076)
Supplement: Supplementary file 1 — Text S1. Additional information regarding sample. Text S2. Additional information regarding statistical analysis. Text S3. Missingness. Text S4. Description of approach used to address potential bias induced by conditioning sample on smoking. Text S5. Description of all observed transitions between nicotine use states. Text S6. Divergence from pre‐registration. Figure S1. Flowchart showing attrition and available sample size. Figure S2. Illustrative example showing how first reported transitions from smoking are identified using synthetic data for five participants. Figure S3. Causal diagram illustrating the relation of the analysis and auxiliary variables with missingness. Figure S4. Correlation matrix showing relationships between variables used in imputation within the eligible sample of participants who responded at the 21 + questionnaire (n = 3,290) used to derive weights related to selection via smoking. Figure S5. Correlation matrix showing relationships between variables used in imputation within the analytic sample of people who smoked in the past 30 days at the 21 + questionnaire (n = 858) used to investigate associations between participant characteristics and transitions from smoking using discrete time subdistribution hazard models. Figure S6. Predictor matrix showing variables included in the imputation model within the eligible sample of participants who responded to the 21 + questionnaire (n = 3,290). All variables used in the selection models were included in each imputation model to ensure consistency in deriving weights related to selection via smoking. Figure S7. Predictor matrix showing variables included in the imputation model within the analytic sample of participants who smoked in the past 30 days at the 21 + questionnaire (n = 858). All variables used in the analysis models were included in each imputation model to ensure consistency in investigating associations between participant characteristics and transitions from smoking using discret [file ADD-120-1804-s001.docx]

**SUPPLEMENTARY MATERIALS: First transitions from smoking to exclusive e-cigarette use, dual use, or no nicotine use and their association with modifiable and sociodemographic factors: Findings from the Avon Longitudinal Study of Parents and Children (ALSPAC) UK birth cohort**

**Corresponding author: Alexandria Andrayas (alex.andrayas@bristol.ac.uk)**

**Contents**

**Supplementary Text**

[Supplementary Text S1. Additional information regarding sample 3](#_Toc189123551)

[Supplementary Text S2. Additional information regarding statistical analysis 3](#_Toc189123552)

[Supplementary Text S3. Missingness 3](#_Toc189123553)

[Supplementary Text S4. Description of approach used to address potential bias induced by conditioning sample on smoking 4](#_Toc189123554)

[Supplementary Text S5. Description of all observed transitions between nicotine use states 5](#_Toc189123555)

[Supplementary Text S6. Divergence from pre-registration 5](#_Toc189123556)

**Supplementary Figures**

[Supplementary Figure S1. Flowchart showing attrition and available sample size 6](#_Toc189123557)

[Supplementary Figure S2. Illustrative example showing how first reported transitions from smoking are identified using synthetic data for five participants 6](#_Toc189123558)

[Supplementary Figure S3. Causal diagram 7](#_Toc189123559)

[Supplementary Figure S4. Correlation matrix showing relationships between variables used in imputation within the eligible sample of participants who responded at the 21+ questionnaire (n=3,290) used to derive weights related to selection via smoking 8](#_Toc189123560)

[Supplementary Figure S5. Correlation matrix showing relationships between variables used in imputation within the analytic sample of people who smoked in the past 30 days at the 21+ questionnaire (n=858) used to investigate associations between participant characteristics and transitions from smoking using discrete time subdistribution hazard models 9](#_Toc189123561)

[Supplementary Figure S6. Predictor matrix showing variables included in the imputation model within the eligible sample of participants who responded to the 21+ questionnaire (n=3,290). All variables used in the selection models were included in each imputation model to ensure consistency in deriving weights related to selection via smoking 10](#_Toc189123562)

[Supplementary Figure S7. Predictor matrix showing variables included in the imputation model within the analytic sample of participants who smoked in the past 30 days at the 21+ questionnaire (n=858). All variables used in the analysis models were included in each imputation model to ensure consistency in investigating associations between participant characteristics and transitions from smoking using discrete-time subdistribution hazard models 11](#_Toc189123563)

[Supplementary Figure S8. Possible collider-conditioning bias when investigating e-cigarette use after conditioning on smoking and approach used to mitigate this bias 12](#_Toc189123564)

[Supplementary Figure S9. Number of participants by self-reported nicotine use and missingness at each timepoint in the analytic sample (n=858) 13](#_Toc189123565)

**Supplementary Tables**

[Supplementary Table S1. Background literature related to the investigated risk factors in the context of smoking tobacco and/or vaping e-cigarettes 14](#_Toc189123566)

[Supplementary Table S2. Checklist of items that should be included in reports of cohort studies 15](#_Toc189123567)

[Supplementary Table S3. Participant characteristics in analytic sample with complete records for baseline confounders and investigated risk factors 16](#_Toc189123568)

[Supplementary Table S4. Predictors (including all analysis variables and those used in imputation) of having a complete record 18](#_Toc189123569)

[Supplementary Table S5. All observed transitions between nicotine use states across 5 timepoints, from ages 21 to 30 years, where nicotine use status when missing was assumed to remain the same as that reported during the previous wave of data collection. The numbers shown in brackets refer to the first reported transition observed following smoking at age 21. 20](#_Toc189123570)

[Supplementary Table S6. Average of all observed transitions between nicotine use states across 5 timepoints, from ages 21 to 30 years, where nicotine use status at all waves of data collection was imputed. The numbers shown in brackets refer to the first reported transition observed following smoking at age 21. 21](#_Toc189123571)

[Supplementary Table S7. Differences in characteristics between participants who did or did not report at least one transition between nicotine use states 22](#_Toc189123572)

[Supplementary Table S8. Differences in characteristics by first reported transitions from smoking in those not loss to follow up, where transitions were derived by assuming transitions did not occur during any missing prior or intermediate reports of nicotine use, excluding any missingness in each characteristic 23](#_Toc189123573)

[Supplementary Table S9. Differences in characteristics by early-life confounders excluding any pairwise missingness 25](#_Toc189123574)

[Supplementary Table S10. Pooled summary statistics for *early-life confounders* and time from sub-distribution discrete time survival analyses, weighted for selection via smoking 26](#_Toc189123575)

[Supplementary Table S11. Pooled summary statistics from sub-distribution discrete time survival analyses, adjusted for early-life confounders, *unweighted* 27](#_Toc189123576)

[Supplementary Table S12. Pooled summary statistics from sub-distribution discrete time survival analyses, *unadjusted*, weighted for selection via smoking 29](#_Toc189123577)

[Supplementary Table S13. Summary statistics from sub-distribution discrete time survival, adjusted for early-life confounders, *complete cases* (n=168), unweighted (as weights rely on imputed data) 30](#_Toc189123578)

Supplementary Text S1. Additional information regarding sample

Pregnant women resident in Avon, UK with expected delivery dates between 1st April 1991 and 31^st^ December 1992 were invited to take part in the study. The initial number of pregnancies enrolled was 14,541, including 14,203 unique mothers, of which 13,988 children were alive at 1 year of age. The total sample size for analyses using any data collected after the age of seven is 15,447 pregnancies, of which 14,901 children were alive at 1 year of age. A total of 14,833 unique women (G0 mothers) were enrolled in ALSPAC as of September 2021. Detailed information has been collected on these women and their offspring (G1) at regular intervals^1–3^. 12,113 G0 partners have been in contact with the study by providing data or formally enrolling when this started in 2010. 3,807 G0 partners are currently enrolled^4,5^.

Repeated measures of e-cigarette use have been collected in ALSPAC from approximately 22 years of age. At each of five timepoints (22, 23, 24, 28, 30 years [y]), nicotine use was characterised based on self-reported smoking in the past 30 days and current e-cigarette use at the time of the questionnaire. At the 30+ questionnaire (at approximately 30y) participants were asked if they had used e-cigarettes in the past 30 days rather than if they were currently using e-cigarettes, as in previous timepoints.

Due to data availability, nicotine use is captured within the 30-day period around each wave of data collection, assuming any observed transitions from smoking occurred within this timeframe and no other non-exclusive smoking event took place outside of it, which may not be plausible in reality.

Supplementary Text S2. Additional information regarding statistical analysis

Time-to-event models were used to explicitly adjust for the varying time of transition when estimating the covariate associations and account for how individuals remain at risk over time. The target quantity is the cumulative incidence of each transition from smoking (to either no nicotine use, exclusive e-cigarette use, or dual use) occurring over time in participants exposed versus unexposed to each risk factor. The analysis model is a discrete-time subdistribution hazard model which is appropriate for our question as it gives insights into how each investigated risk factor affects the probability of each transition, while accounting for competing transitions and properly weighting their contribution rather than treating them as censored. Analyses are adjusted for early-life confounders (sex, ethnicity household income, parental smoking).

The subdistribution hazard function is defined as the instantaneous rate of occurrence, at any time, of the event of interest (where this denotes a particular initial transition from smoking), in participants who have not yet experienced an event of that type (meaning they either experienced no event, or a competing event i.e. any other transition from smoking). Subdistribution hazard models offer inferences about the relative magnitude and direction of the effect of covariates on the cumulative incidence function (CIF) for the event of interest but do not necessarily convey the absolute magnitude of this effect, nor can magnitudes between different subdistribution hazard models be compared. Therefore discussion of these results focuses on the relative importance and direction of effects using subdistribution hazard ratios (SHR) and 95% confidence intervals (CI).

Supplementary Text S3. Missingness

Many of the covariates used in the analysis have been shown to be associated with missingness and so data are not missing completely at random (MCAR). In addition attrition is a known issue in ALSPAC^6,7^ and we expected missingness to be associated with the outcome. Therefore a complete records analysis may yield biased estimates of the effects of risk factors on first transitions from smoking. However, there are also many strong predictors of missing data available at previous and later waves of data collection, where earlier measures tend to be more complete. In this case multiple imputation (MI) using these auxiliary variables may reduce bias and improve precision over a complete records analysis. Therefore MI was used in the primary analysis but complete records analysis was still conducted and used as a sensitivity analysis as it relies on different assumption about the missing data. A causal diagram is shown in Supplementary Figure S3 to illustrate that we expect missingness to depend on the outcome of interest, transitions from smoking, as well as the risk factors of interest and included confounders, and that there are auxiliary variables that are both associated with missingness and with the incomplete variables used in the analysis.

Table 1 shows both the proportion of missing data for all variables in the analysis models, and the observed characteristics for all participants. The observed characteristics for the participants with complete records is shown in Supplementary Table S3. Predictors of missingness are assessed in Supplementary Table S4. In this sample only 20% (n = 173) of the sample, meaning the 859 participants who reported smoking in the past month at age 21, had complete data on all baseline confounders and main exposures. 11% (n=95) also had complete data on all 5 nicotine use status measures. A small number (n=38) of participants included in the complete case analysis were missing an intermediate nicotine status measure prior to their first observed transition. For these participants, in the complete case analysis only, we assumed the transition occurred at the time of the first observed change in smoking status. Participants with complete data on all baseline confounders and main exposures differed compared to those with incomplete data by many of the included variables. The variables most associated with missingness in the present study include parental smoking at ages 2 and 12, smoking more frequently at age 21, lower educational attainment at age 18, less frequent binge drinking at age 18, peer smoking and other drug use at age 16, and being a parent at ages 21 and 22.

MI by chained equations via the mice R package was used to impute any missing data using 100 imputations with 25 iterations. Inclusion of auxiliary variables as predictors of other variables in the imputation model was based on a minimum correlation threshold of 0.1. Correlation and predictor matrices are shown in Supplementary Figures S4-7. The first reported transitions from smoking were passively imputed given their dependence on the repeated measures of nicotine use status. This refers to the first non-exclusive smoking event reported during any of the 5 waves of data collection following the 21+ questionnaire, where the time to this event was defined as the difference in approximate total years between the 21+ questionnaire and the questionnaire/clinic at which the event was reported. For example, suppose Participant A reported exclusively smoking at ages 22, 23, and 24, but at age 28 they reported dual use (both smoking and e-cigarette use). In this case, their first reported transition from smoking would be inferred as ‘dual use’ occurring 7 years after they initially reported smoking at age 21. By using passive imputation, we ensured that each participant had a defined first transition event while maintaining consistency with their available nicotine use data across multiple timepoints. This approach helped to minimize bias due to missing data while preserving the temporal ordering of transitions from smoking.

Results from both complete cases records and MI analyses showed some discrepancies. When analyses were limited to complete cases there was no association of parenthood at age 21 with the probability of transitioning to no nicotine use, but young parents did appear more likely to first report dual use. Unweighted complete case analyses also showed no association of smoking frequency with the probability of transitioning to exclusive e-cigarette use, nor BMI and peer smoking with first transitions to dual use. However, these results are likely more biased than those described above due to missing data. Although missing data had some impact on the inference of results, the inferences were the same and/or similar for the strongest results and so improved confidence that there is little risk of bias due to missing data.

**Supplementary Text S4. Description of approach used to address potential bias induced by conditioning sample on smoking**

Collider bias occurs when an analysis controls for, stratifies on, or selects its sample based on a variable that is caused by both the exposure and the outcome. This distorts the association between the exposure and outcome and can lead to incorrect inferences from effect estimates^8^. In this case there are shared risk factors related to both e-cigarette and smoking (Supplementary Figure S8A). Therefore when selecting our sample to just those who smoke, we may induce a negative association between smoking risk factors and vaping and incorrectly conclude that risk factors for smoking are protective against vaping even though in reality they are unrelated^9^.

To mitigate this bias a two-step approach was utilised where first a logistic regression model is fitted to predict smoking in the past 30 days at age 21 (Supplementary Figure S8B). Secondly the regression model for e-cigarette use, estimated on the selected subsample of participants who smoke, is weighted using inverse predicted probabilities derived from the first model (Supplementary Figure S8C) to reduce bias^10^. The predictors included in the selection model were the four baseline confounders included in analysis models (sex, ethnicity, household income at age 11, parent daily smoking at age 12), plus parental mental health at age 12, peer smoking, substance use (including alcohol, cannabis, and other drugs separately), and educational aspirations at age 16, and exercise frequency, BMI and depressive symptoms at age 18.

**References**

1. Boyd A, Golding J, Macleod J, et al. Cohort Profile: The ‘Children of the 90s’—the index offspring of the Avon Longitudinal Study of Parents and Children. *Int J Epidemiol*. 2013;42(1):111-127. doi:10.1093/ije/dys064

2. Fraser A, Macdonald-Wallis C, Tilling K, et al. Cohort Profile: The Avon Longitudinal Study of Parents and Children: ALSPAC mothers cohort. *Int J Epidemiol*. 2013;42(1):97-110. doi:10.1093/ije/dys066

3. Northstone K, Lewcock M, Groom A, et al. The Avon Longitudinal Study of Parents and Children (ALSPAC): an update on the enrolled sample of index children in 2019. *Wellcome Open Res*. 2019;4:51. doi:10.12688/wellcomeopenres.15132.1

4. Major-Smith D, Heron J, Fraser A, Lawlor DA, Golding J, Northstone K. The Avon Longitudinal Study of Parents and Children (ALSPAC): a 2022 update on the enrolled sample of mothers and the associated baseline data. *Wellcome Open Res*. 2022;7:283. doi:10.12688/wellcomeopenres.18564.1

5. Northstone K, Ben-Shlomo Y, Teyhan A, et al. The Avon Longitudinal Study of Parents and children ALSPAC G0 ... *Wellcome Open Res*. Published online July 2023. doi:10.12688/wellcomeopenres.18782.2

6. Howe LD, Tilling K, Galobardes B, Lawlor DA. Loss to follow-up in cohort studies: bias in estimates of socioeconomic inequalities. *Epidemiol Camb Mass*. 2013;24(1):1-9. doi:10.1097/EDE.0b013e31827623b1

7. Taylor AE, Jones HJ, Sallis H, et al. Exploring the association of genetic factors with participation in the Avon Longitudinal Study of Parents and Children. *Int J Epidemiol*. 2018;47(4):1207-1216. doi:10.1093/ije/dyy060

8. Digitale JC, Martin JN, Glidden DV, Glymour MM. Key concepts in clinical epidemiology: collider-conditioning bias. *J Clin Epidemiol*. 2023;161:152-156. doi:10.1016/j.jclinepi.2023.07.004

9. Tattan-Birch H, Marsden J, West R, Gage SH. Assessing and addressing collider bias in addiction research: the curious case of smoking and COVID-19. *Addiction*. 2021;116(5):982-984. doi:10.1111/add.15348

10. Breen R, Ermisch J. Using Inverse Probability Weighting to Address Post-Outcome Collider Bias. *Sociol Methods Res*. 2024;53(1):5-27. doi:10.1177/00491241211043131

Supplementary Text S5. Description of all observed transitions between nicotine use states

In the analytical sample of 859 participants who smoked in the past month at age 21, and when ignoring missing intermediate nicotine use status measures, 252 participants, including 55 lost to follow-up and 197 who only reported exclusive smoking, showed no changes in nicotine use status. There was only one observed change in nicotine use status for most participants (n=323). There were 146, 104, 29 and 4 participants who reported 2, 3, 4, or 5 changes in nicotine use status respectively. Supplementary Tables S5-6 show the number of transitions between all nicotine use states observed between the ages of 21 to 30 in the analytical sample. This also shows that there were too few subsequent transitions (i.e. after the first transition from smoking) for them to be properly modelled. Other than the investigated first-reported transitions from smoking, the subsequent transitions most observed were from no nicotine use, or dual use, back to exclusive smoking. This includes transitions where the participant may have relapsed back to smoking but then reverted to the nicotine use status reported in their first transition.

**Supplementary Text S6. Divergence from pre-registration**

The pre-registration for this study (<https://osf.io/u6g4s/>) stated that logistic regression and cause-specific survival analyses were to be used to investigate associations between participant characteristics and first reported transitions from smoking. However, these were not taken forward given that logistic regression does not consider the time-to-event, and cause-specific survival analysis does not consider the occurrence of competing events. As the investigated events are transient and not absorbing, subdistribution survival analysis then better fit the research question.

The pre-registration also stated that multi-state models were to be fitted to investigate all possible transitions, not just the first reported transitions from smoking, however this was not possible due to limited sample size and statistical power.


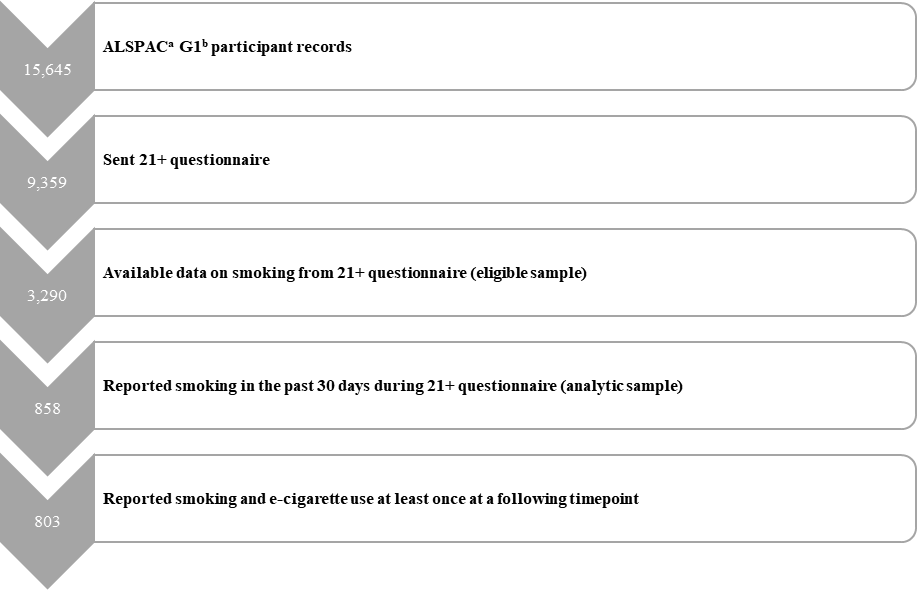


**^a^Avon Longitudinal Study of Parents and Children; ^b^ Offspring of women resident in Avon, UK with expected delivery dates between 1st April 1991 and 31st December 1992 who took part in the study**

Supplementary Figure S1. Flowchart showing attrition and available sample size


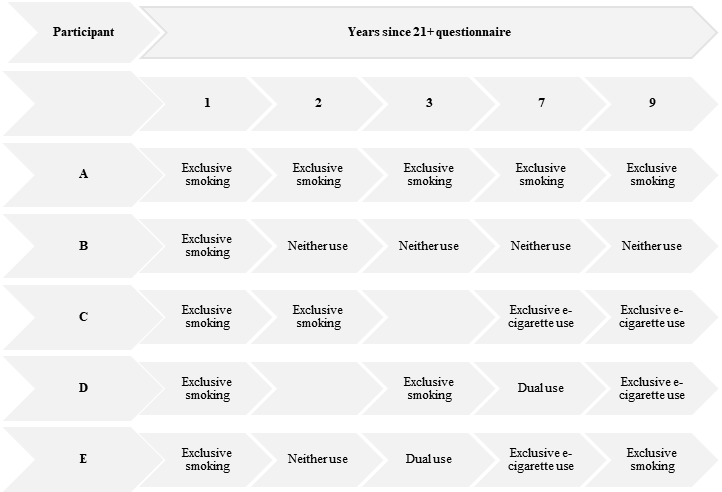


**Each row (light grey) represents synthetic data for each of five (A-E) participants. Each column (light grey) represents synthetic reported nicotine use by each participant at each of five timepoints (1, 2, 3, 7, and 9 years following the 21+ questionnaire). The dark grey boxes represent the derived first reported transition from smoking for each participant. For example, participant A only reported exclusively smoking and so did not transition. Participant D reported exclusively smoking at all timepoints up to 7 years, and then reported dual use (of cigarettes and e-cigarettes) and so their first transition from smoking would be to dual use.**


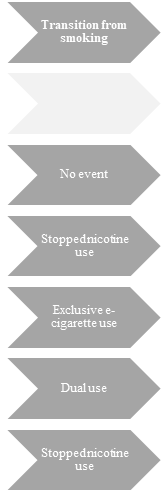


Supplementary Figure S2. Illustrative example showing how first reported transitions from smoking are identified using synthetic data for five participants

**
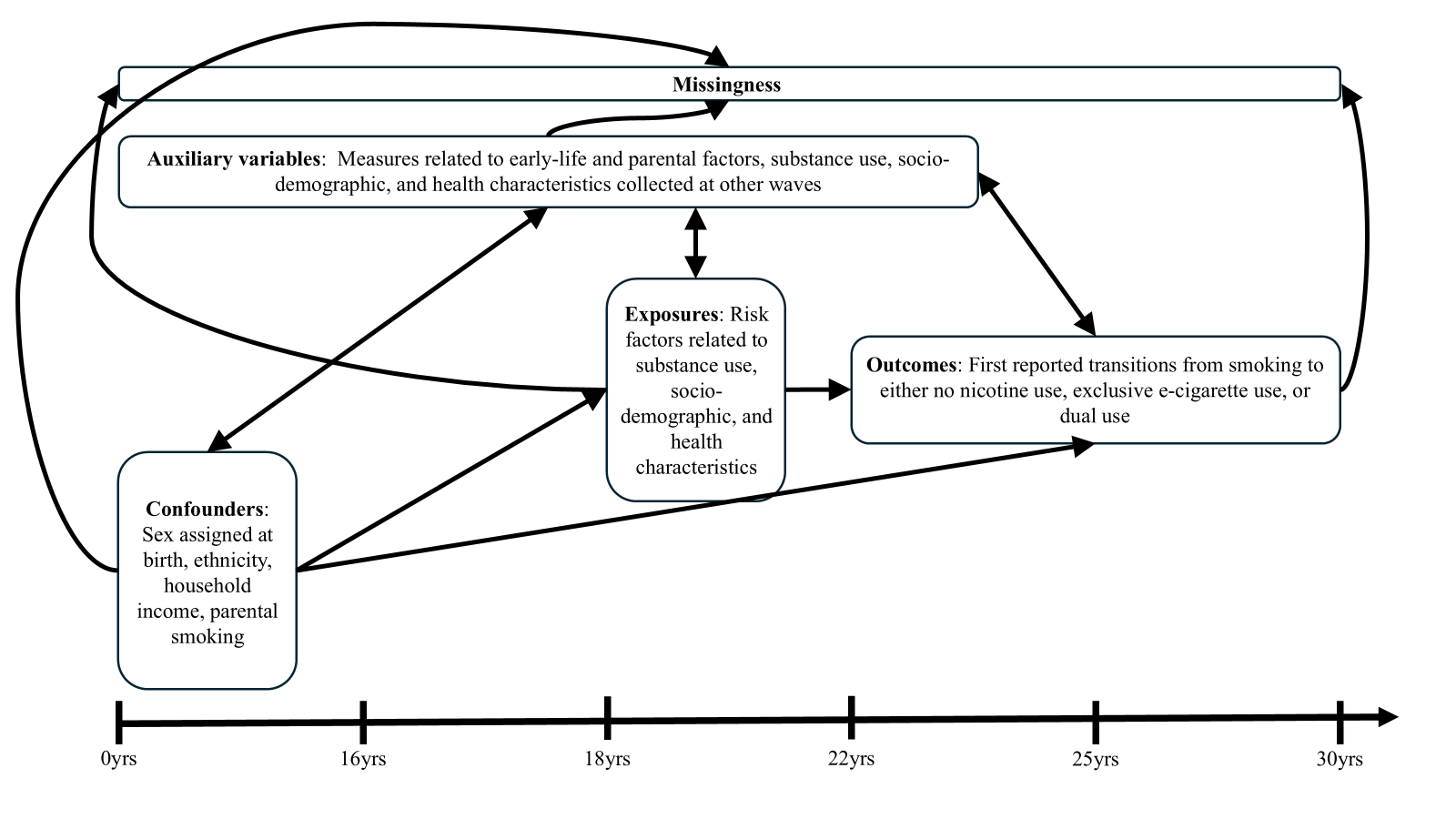
**

Supplementary Figure S3. Causal diagram illustrating the relation of the analysis and auxiliary variables with missingness


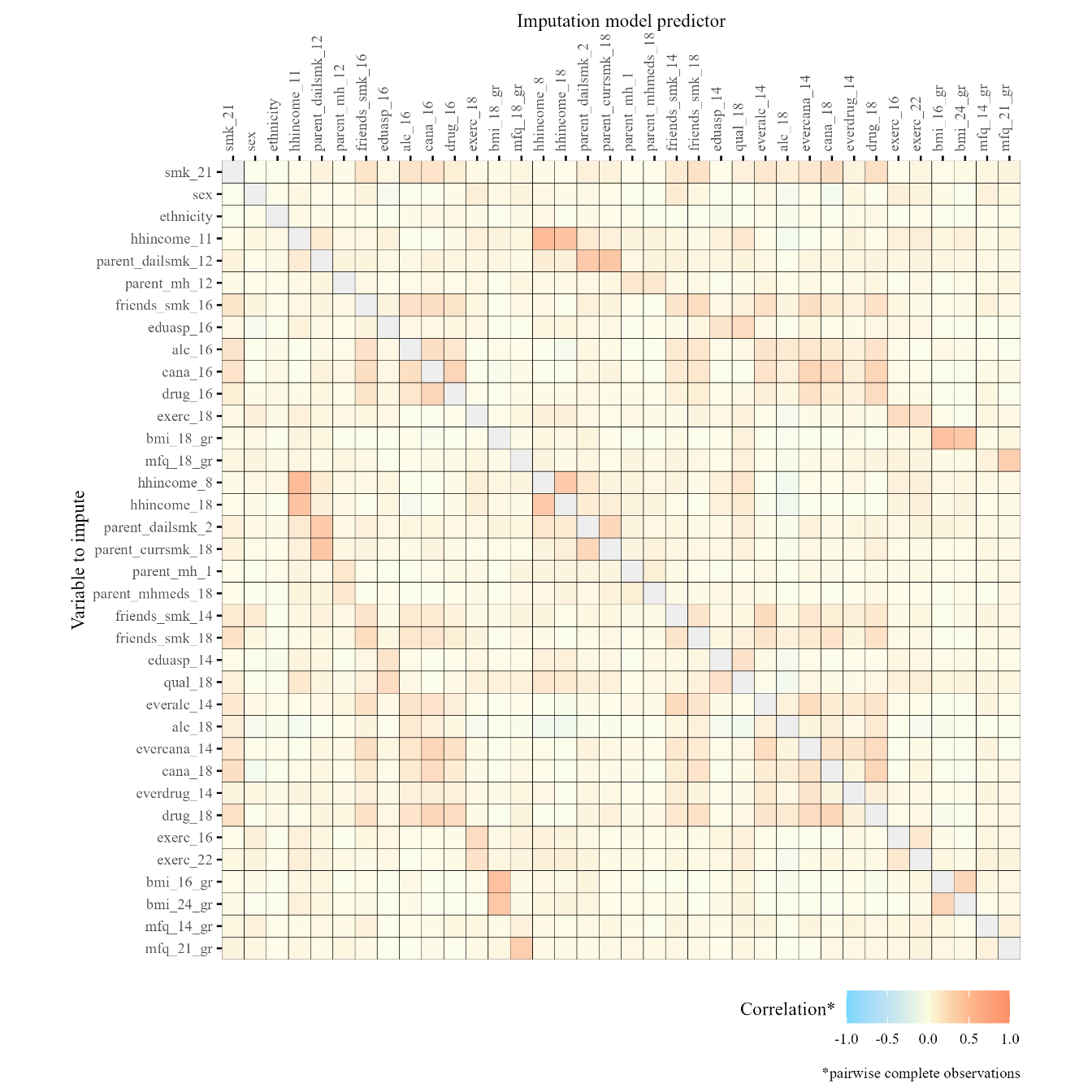


Supplementary Figure S4. Correlation matrix showing relationships between variables used in imputation within the eligible sample of participants who responded at the 21+ questionnaire (n=3,290) used to derive weights related to selection via smoking


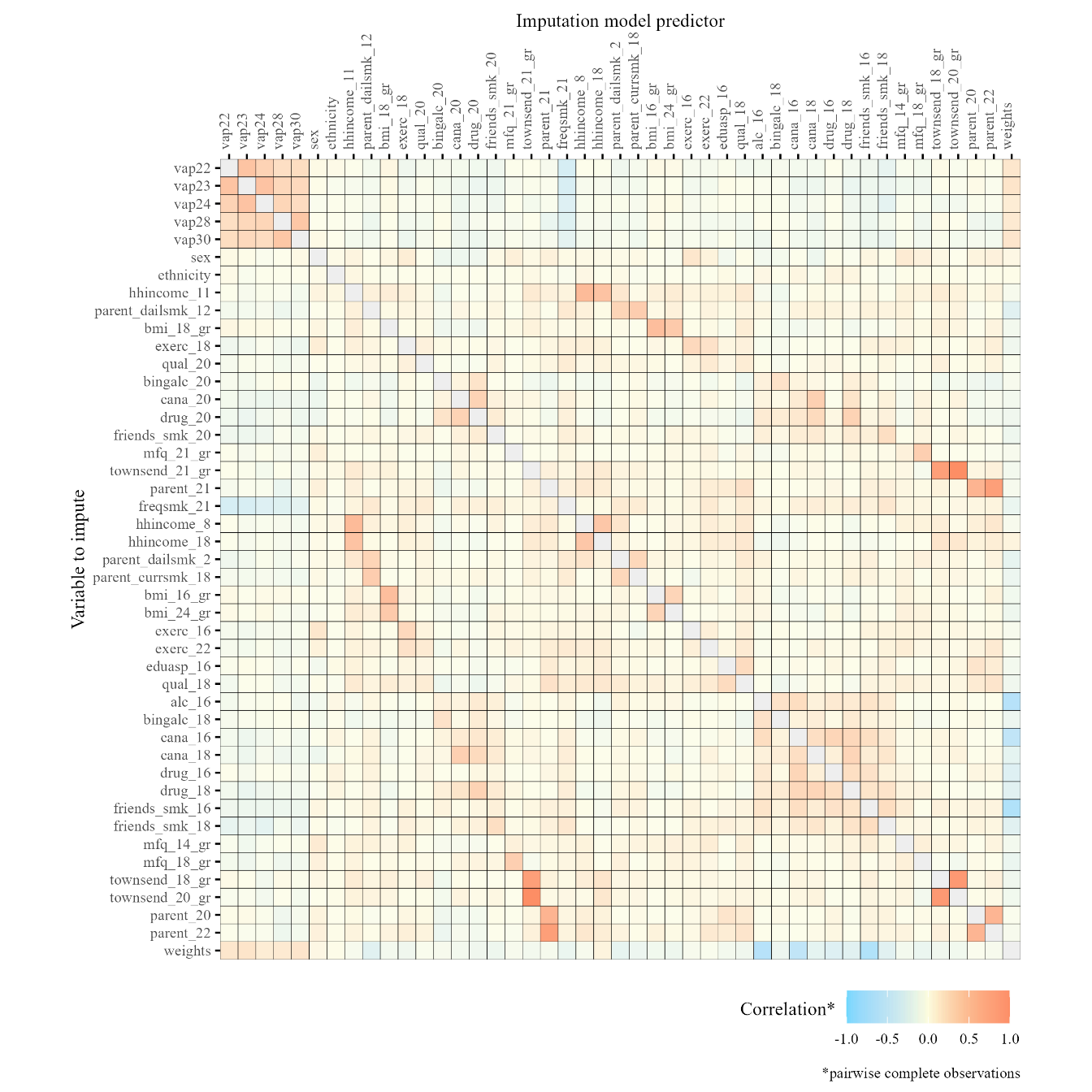


Supplementary Figure S5. Correlation matrix showing relationships between variables used in imputation within the analytic sample of people who smoked in the past 30 days at the 21+ questionnaire (n=858) used to investigate associations between participant characteristics and transitions from smoking using discrete time subdistribution hazard models


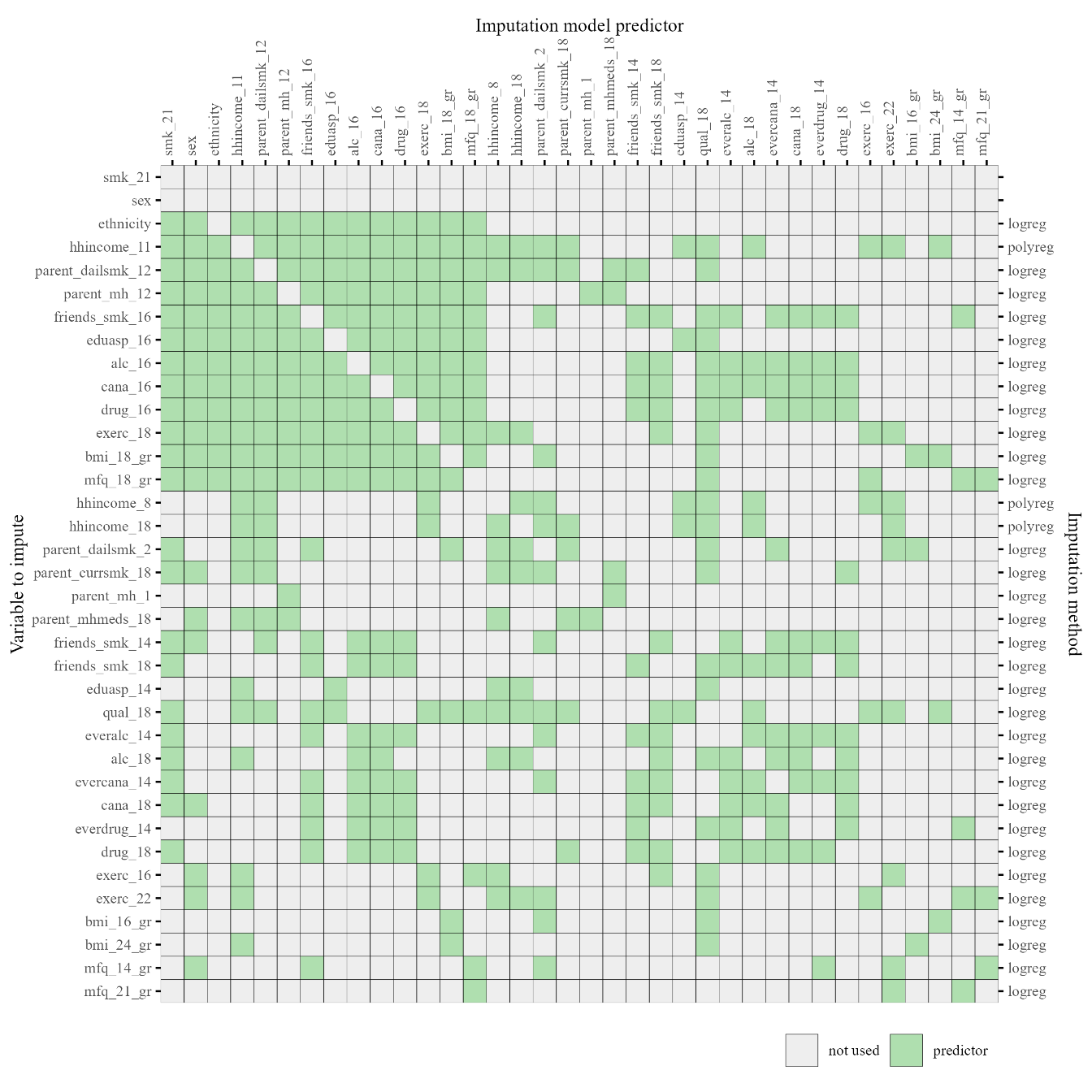


Supplementary Figure S6. Predictor matrix showing variables included in the imputation model within the eligible sample of participants who responded to the 21+ questionnaire (n=3,290). All variables used in the selection models were included in each imputation model to ensure consistency in deriving weights related to selection via smoking


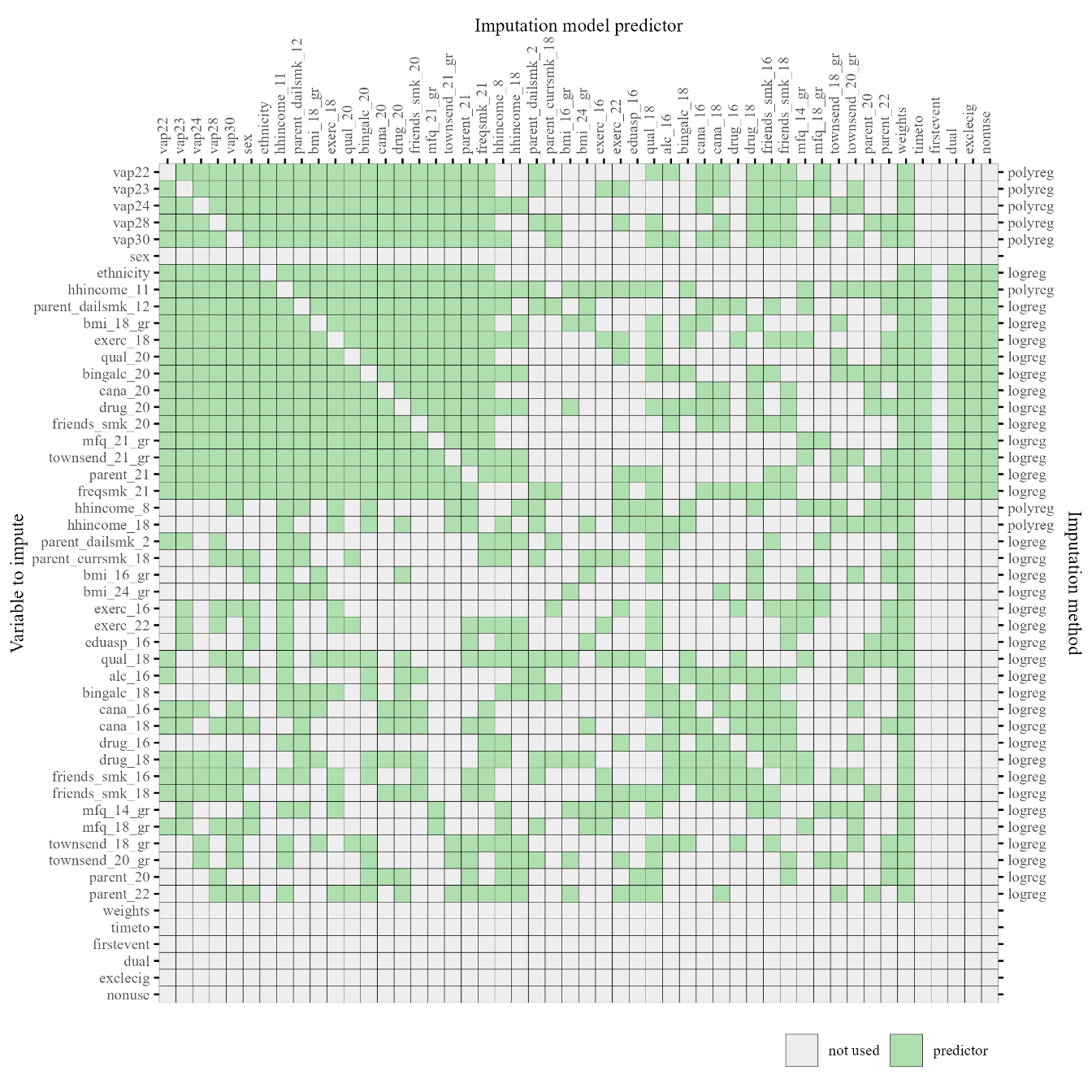


Supplementary Figure S7. Predictor matrix showing variables included in the imputation model within the analytic sample of participants who smoked in the past 30 days at the 21+ questionnaire (n=858). All variables used in the analysis models were included in each imputation model to ensure consistency in investigating associations between participant characteristics and transitions from smoking using discrete-time subdistribution hazard models


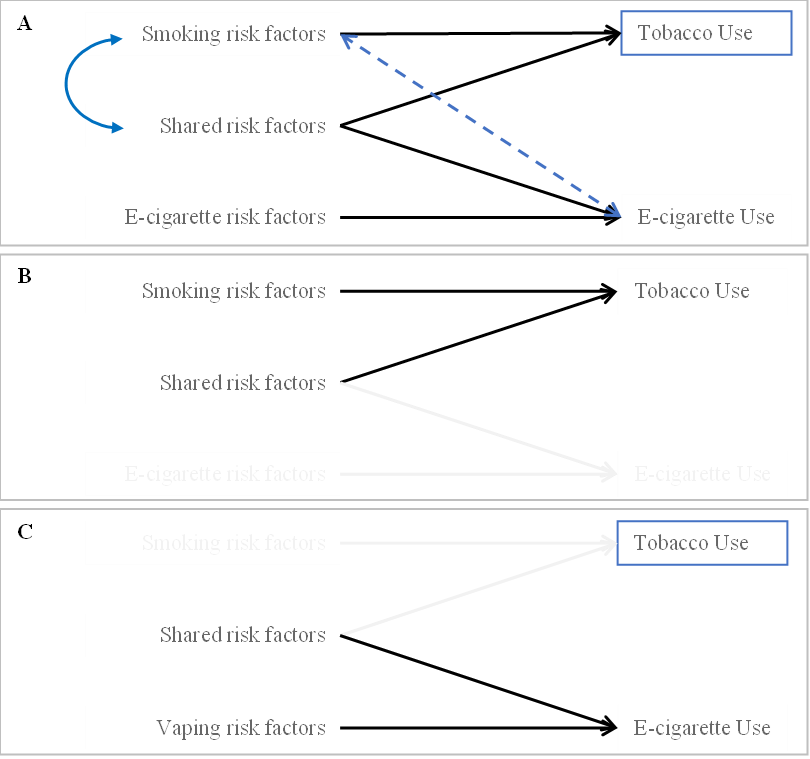


Supplementary Figure S8. Possible collider-conditioning bias when investigating e-cigarette use after conditioning on smoking and approach used to mitigate this bias


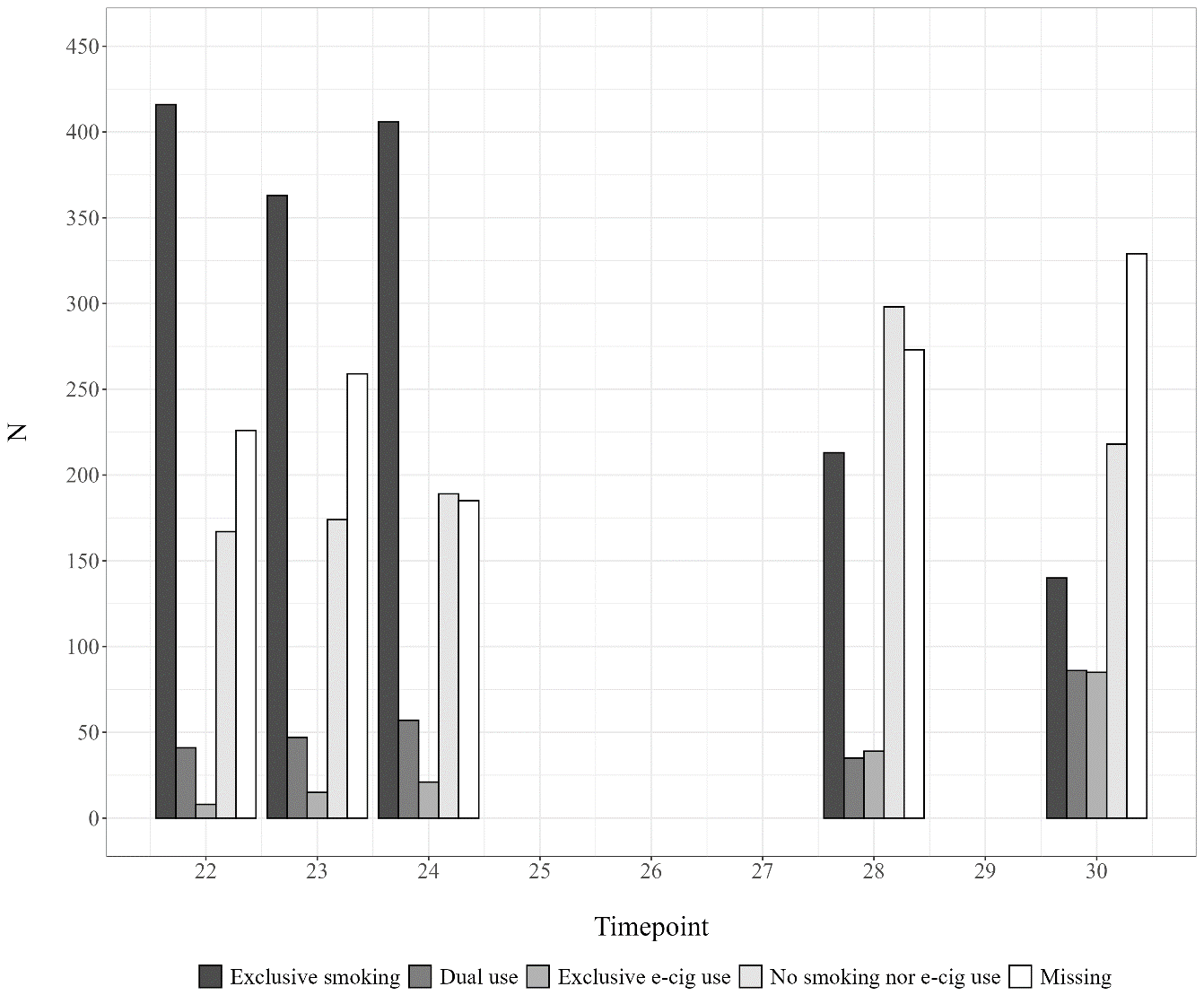


Supplementary Figure S9. Number of participants by self-reported nicotine use and missingness at each timepoint in the analytic sample (n=858)

Supplementary Table S1. Background literature related to the investigated risk factors in the context of smoking tobacco and/or vaping e-cigarettes

| **Risk factor** | **Literature** | **Key findings** |
| --- | --- | --- |
| **Smoking frequency** | Brown et al., 2014  Pearson et al., 2015 | Individuals who smoke more cigarettes, and therefore perhaps more nicotine dependant, are more likely to use e-cigarettes. |
| **Alcohol** | van Amsterdam and van den Brink, 2023 | There is a co-occurrence of tobacco smoking and alcohol consumption, and being a non-drinker is associated with a more successful smoking cessation outcome. |
| **Cannabis use** | Hindocha et al, 2016; 2021  Gravely et al., 2022  Rice et al., 2022 | Cannabis and tobacco are linked due to their co-use in the UK but previous findings relating to the links between cannabis and e-cigarette use have been mixed. |
| **Other drug use** | Teesson et al., 2012 | High prevalence of comorbidity between smoking and illicit drug use. |
| **Peer smoking** | Amin et al., 2020 | People with more social connections who smoke are more likely to use e-cigarettes. |
| **Education** | Wilson and Wang, 2017 | Adults with higher levels of education are more likely to use e-cigarettes. |
| **Early parenthood** | Jefferis et al., 2004  Nabi-Burza et al., 2019 | Early parenthood has been linked to persistent smoking but parents who dual use may contemplate smoking cessation more than parents who only smoke cigarettes. |
| **Neighbourhood deprivation** | Pearce et al., 2011 | Higher smoking prevalence is associated with socio-spatial inequalities. |
| **BMI** | Lanza et al., 2017 | Individuals who have a higher BMI are more likely to use substances (tobacco/nicotine, cannabis, alcohol), but overweight young adults may be at higher risk than obese young adults. |
| **Exercise** | Pokhrel et al., 2020  Kujala et al., 2007 | Higher levels of physical activity have been linked to higher e-cigarette use in young adults, but lower cigarette smoking. |
| **Depression** |  | Self-reported depression was associated with increased odds of ploy-use between substances including tobacco, e-cigarettes and cannabis. |

Supplementary Table S2. Checklist of items that should be included in reports of cohort studies

|  | Item | Recommendation | Included |
| --- | --- | --- | --- |
| Title and abstract | 1 | (*a*) Indicate the study’s design with a commonly used term in the title or the abstract | ✓ |
|  |  | (*b*) Provide in the abstract an informative and balanced summary of what was done and what was found | ✓ |
| Introduction | | |  |
| Background/rationale | 2 | Explain the scientific background and rationale for the investigation being reported | ✓ |
| Objectives | 3 | State specific objectives, including any prespecified hypotheses | ✓ |
| Methods | | |  |
| Study design | 4 | Present key elements of study design early in the paper | ✓ |
| Setting | 5 | Describe the setting, locations, and relevant dates, including periods of recruitment, exposure, follow-up, and data collection | ✓ |
| Participants | 6 | (*a*) Give the eligibility criteria, and the sources and methods of selection of participants. Describe methods of follow-up | ✓ |
|  |  | (*b*) For matched studies, give matching criteria and number of exposed and unexposed | NA |
| Variables | 7 | Clearly define all outcomes, exposures, predictors, potential confounders, and effect modifiers. Give diagnostic criteria, if applicable | ✓ |
| Data sources/ measurement | 8* | For each variable of interest, give sources of data and details of methods of assessment (measurement). Describe comparability of assessment methods if there is more than one group | ✓ |
| Bias | 9 | Describe any efforts to address potential sources of bias | ✓ |
| Study size | 10 | Explain how the study size was arrived at | ✓ |
| Quantitative variables | 11 | Explain how quantitative variables were handled in the analyses. If applicable, describe which groupings were chosen and why | ✓ |
| Statistical methods | 12 | (*a*) Describe all statistical methods, including those used to control for confounding | ✓ |
|  |  | (*b*) Describe any methods used to examine subgroups and interactions | NA |
|  |  | (*c*) Explain how missing data were addressed | ✓ |
|  |  | (*d*) If applicable, explain how loss to follow-up was addressed | ✓ |
|  |  | (*e*) Describe any sensitivity analyses | ✓ |
| Results | | |  |
| Participants | 13* | (a) Report numbers of individuals at each stage of study—eg numbers potentially eligible, examined for eligibility, confirmed eligible, included in the study, completing follow-up, and analysed | ✓ |
|  |  | (b) Give reasons for non-participation at each stage | ✓ |
|  |  | (c) Consider use of a flow diagram | ✓ |
| Descriptive data | 14* | (a) Give characteristics of study participants (eg demographic, clinical, social) and information on exposures and potential confounders | ✓ |
|  |  | (b) Indicate number of participants with missing data for each variable of interest | ✓ |
|  |  | (c) Summarise follow-up time (eg, average and total amount) | ✓ |
| Outcome data | 15* | Report numbers of outcome events or summary measures over time | ✓ |
| Main results | 16 | (*a*) Give unadjusted estimates and, if applicable, confounder-adjusted estimates and their precision (eg, 95% confidence interval). Make clear which confounders were adjusted for and why they were included | ✓ |
|  |  | (*b*) Report category boundaries when continuous variables were categorized | ✓ |
|  |  | (*c*) If relevant, consider translating estimates of relative risk into absolute risk for a meaningful time period | NA |
| Other analyses | 17 | Report other analyses done—eg analyses of subgroups and interactions, and sensitivity analyses | ✓ |
| Discussion | | |  |
| Key results | 18 | Summarise key results with reference to study objectives | ✓ |
| Limitations | 19 | Discuss limitations of the study, taking into account sources of potential bias or imprecision. Discuss both direction and magnitude of any potential bias | ✓ |
| Interpretation | 20 | Give a cautious overall interpretation of results considering objectives, limitations, multiplicity of analyses, results from similar studies, and other relevant evidence | ✓ |
| Generalisability | 21 | Discuss the generalisability (external validity) of the study results | ✓ |
| Other information | | |  |
| Funding | 22 | Give the source of funding and the role of the funders for the present study and, if applicable, for the original study on which the present article is based | ✓ |

Supplementary Table S3. Participant characteristics in analytic sample with complete records for baseline confounders and investigated risk factors

| **Characteristic** | **N = 173***^1^* |
| --- | --- |
| **Sex assigned at birth** |  |
| *Male* | 66 (38%) |
| *Female* | 107 (62%) |
| **Ethnicity** |  |
| *White* | 169 (98%) |
| *BAME** | 4 (2.3%) |
| **Average household income, GBP per week (11y)** |  |
| *560+* | 78 (45%) |
| *430-559* | 36 (21%) |
| *240-429* | 46 (27%) |
| *<240* | 13 (7.5%) |
| **Parental smoking (12y)** |  |
| *No* | 147 (85%) |
| *Yes* | 26 (15%) |
| **Smoking frequency (21y)** |  |
| *Occasional* | 84 (49%) |
| *Weekly or more* | 89 (51%) |
| **Binge drinking, six or more units (20y)** |  |
| *Never, monthly, or less* | 89 (51%) |
| *Weekly or more* | 84 (49%) |
| **Cannabis use (20y)** |  |
| *Never, less than monthly, or not in past year* | 133 (77%) |
| *Monthly or more* | 40 (23%) |
| **Drug use (20y)** |  |
| *No* | 99 (57%) |
| *Yes* | 74 (43%) |
| **Peer smoking (20y)** |  |
| *None, a few, or some* | 70 (40%) |
| *Most or all* | 103 (60%) |
| **Educational qualifications (20y)** |  |
| *Degree-level* | 40 (23%) |
| *A-level or lower/Other* | 133 (77%) |
| **Is a parent (21y)** |  |
| *No* | 168 (97%) |
| *Yes* | 5 (2.9%) |
| **G1 Townsend deprivation score, quintiles (21y)** |  |
| *Least deprived* | 73 (42%) |
| *More deprived* | 100 (58%) |
| **BMI (18y)** |  |
| *<25* | 141 (82%) |
| *>=25* | 32 (18%) |
| **Frequency of exercise, past year (18y)** |  |
| *Weekly or more* | 116 (67%) |
| *Less than weekly* | 57 (33%) |
| **Mood and Feelings Questionnaire scores (21y)** |  |
| *<12* | 144 (83%) |
| *>=12* | 29 (17%) |
| *^1^*n (%) | |

Supplementary Table S4. Predictors (including all analysis variables and those used in imputation) of having a complete record

| **Characteristic** | **N** | **OR***^1^* | **95% CI***^1^* | **p-value** |
| --- | --- | --- | --- | --- |
| **First reported transition** | 803 |  |  |  |
| *None reported* |  | — | — |  |
| *Dual use* |  | 1.62 | 0.95, 2.78 | 0.080 |
| *Exclusive e-cigarette use* |  | 1.45 | 0.67, 3.00 | 0.3 |
| *Non-use* |  | 1.68 | 1.08, 2.68 | 0.025 |
| **Time to first transition** | 803 | 0.99 | 0.94, 1.05 | 0.7 |
| **Sex assigned at birth** | 858 |  |  |  |
| *Male* |  | — | — |  |
| *Female* |  | 0.95 | 0.67, 1.35 | 0.8 |
| **Ethnicity** | 788 |  |  |  |
| *White* |  | — | — |  |
| *BAME** |  | 0.43 | 0.10, 1.25 | 0.2 |
| **Average household income, GBP per week (11y)** | 651 |  |  |  |
| *560+* |  | — | — |  |
| *430-559* |  | 0.87 | 0.54, 1.37 | 0.6 |
| *240-429* |  | 0.84 | 0.54, 1.30 | 0.4 |
| *<240* |  | 0.59 | 0.29, 1.11 | 0.11 |
| **Parental smoking (12y)** | 721 |  |  |  |
| *No* |  | — | — |  |
| *Yes* |  | 0.50 | 0.31, 0.78 | 0.003 |
| **BMI (18y)** | 605 |  |  |  |
| *<25* |  | — | — |  |
| *>=25* |  | 0.72 | 0.46, 1.12 | 0.2 |
| **Frequency of exercise, past year (18y)** | 483 |  |  |  |
| *Weekly or more* |  | — | — |  |
| *Less than weekly* |  | 0.89 | 0.60, 1.32 | 0.6 |
| **Educational qualifications (20y)** | 647 |  |  |  |
| *Degree-level* |  | — | — |  |
| *A-level or lower/Other* |  | 0.82 | 0.54, 1.26 | 0.4 |
| **Binge drinking, six or more units (20y)** | 630 |  |  |  |
| *Never, monthly, or less* |  | — | — |  |
| *Weekly or more* |  | 1.26 | 0.89, 1.80 | 0.2 |
| **Cannabis use (20y)** | 643 |  |  |  |
| *Never, less than monthly, or not in past year* |  | — | — |  |
| *Monthly or more* |  | 0.90 | 0.59, 1.36 | 0.6 |
| **Drug use (20y)** | 613 |  |  |  |
| *No* |  | — | — |  |
| *Yes* |  | 0.97 | 0.68, 1.39 | 0.9 |
| **Peer smoking (20y)** | 640 |  |  |  |
| *None, a few, or some* |  | — | — |  |
| *Most or all* |  | 1.02 | 0.71, 1.46 | >0.9 |
| **Mood and Feelings Questionnaire scores (21y)** | 828 |  |  |  |
| *<12* |  | — | — |  |
| *>=12* |  | 0.70 | 0.44, 1.08 | 0.11 |
| **G1 Townsend deprivation score, quintiles (21y)** | 835 |  |  |  |
| *Least deprived* |  | — | — |  |
| *More deprived* |  | 0.68 | 0.48, 0.97 | 0.030 |
| **Is a parent (21y)** | 849 |  |  |  |
| *No* |  | — | — |  |
| *Yes* |  | 0.23 | 0.07, 0.56 | 0.005 |
| **Smoking frequency (21y)** | 853 |  |  |  |
| *Occasional* |  | — | — |  |
| *Weekly or more* |  | 0.53 | 0.38, 0.75 | <0.001 |
| **Average household income, GBP per week (8y)** | 669 |  |  |  |
| *400+* |  | — | — |  |
| *300-399* |  | 0.58 | 0.33, 0.98 | 0.048 |
| *200-299* |  | 0.89 | 0.52, 1.47 | 0.6 |
| *<200* |  | 0.81 | 0.42, 1.49 | 0.5 |
| **Average household income, GBP per month (18y)** | 458 |  |  |  |
| *3,400+* |  | — | — |  |
| *2,400-3,399* |  | 0.68 | 0.39, 1.16 | 0.2 |
| *1,550-2,399* |  | 0.94 | 0.55, 1.57 | 0.8 |
| *<1,550* |  | 0.77 | 0.41, 1.39 | 0.4 |
| **Parental smoking (2y)** | 755 |  |  |  |
| *No* |  | — | — |  |
| *Yes* |  | 0.61 | 0.41, 0.90 | 0.015 |
| **Parental smoking (18y)** | 606 |  |  |  |
| *No* |  | — | — |  |
| *Yes* |  | 0.75 | 0.44, 1.22 | 0.3 |
| **BMI (16y)** | 623 |  |  |  |
| *<25* |  | — | — |  |
| *>=25* |  | 0.54 | 0.28, 0.98 | 0.053 |
| **BMI (24y)** | 548 |  |  |  |
| *<25* |  | — | — |  |
| *>=25* |  | 0.89 | 0.59, 1.32 | 0.6 |
| **Frequency of exercise, past year (16y)** | 664 |  |  |  |
| *Weekly or more* |  | — | — |  |
| *Less than weekly* |  | 0.86 | 0.55, 1.30 | 0.5 |
| **Frequency of exercise, past year (22y)** | 633 |  |  |  |
| *Weekly or more* |  | — | — |  |
| *Less than weekly* |  | 0.93 | 0.64, 1.34 | 0.7 |
| **Plans after Year 11 (16y)** | 644 |  |  |  |
| *Stay in education* |  | — | — |  |
| *Leave education* |  | 0.41 | 0.12, 1.05 | 0.095 |
| **Educational qualifications (18y)** | 492 |  |  |  |
| *A-level* |  | — | — |  |
| *GCSE or lower/Other* |  | 0.42 | 0.27, 0.65 | <0.001 |
| **Alcohol consumption (16y)** | 622 |  |  |  |
| *Never, or less than weekly* |  | — | — |  |
| *Weekly or more* |  | 0.71 | 0.47, 1.06 | 0.10 |
| **Binge drinking, six or more units (18y)** | 684 |  |  |  |
| *Never, monthly, or less* |  | — | — |  |
| *Weekly or more* |  | 1.71 | 1.19, 2.45 | 0.004 |
| **Cannabis use (16y)** | 615 |  |  |  |
| *Never, once or twice, or used to* |  | — | — |  |
| *Sometimes, weekly, or more* |  | 0.53 | 0.30, 0.89 | 0.021 |
| **Cannabis use (18y)** | 621 |  |  |  |
| *Never, or less than monthly* |  | — | — |  |
| *Monthly or more* |  | 0.68 | 0.41, 1.08 | 0.11 |
| **Drug use (16y)** | 583 |  |  |  |
| *No* |  | — | — |  |
| *Yes* |  | 0.18 | 0.04, 0.51 | 0.005 |
| **Drug use (18y)** | 689 |  |  |  |
| *No* |  | — | — |  |
| *Yes* |  | 0.81 | 0.53, 1.21 | 0.3 |
| **Peer smoking (16y)** | 622 |  |  |  |
| *None, one, or some* |  | — | — |  |
| *Most or all* |  | 0.50 | 0.33, 0.75 | 0.001 |
| **Peer smoking (18y)** | 488 |  |  |  |
| *None, one, or some* |  | — | — |  |
| *Most or all* |  | 0.89 | 0.60, 1.32 | 0.6 |
| **Mood and Feelings Questionnaire scores (14y)** | 650 |  |  |  |
| *<12* |  | — | — |  |
| *>=12* |  | 0.88 | 0.49, 1.50 | 0.6 |
| **Mood and Feelings Questionnaire scores (18y)** | 627 |  |  |  |
| *<12* |  | — | — |  |
| *>=12* |  | 0.55 | 0.32, 0.89 | 0.020 |
| **G1 Townsend deprivation score, quintiles (18y)** | 489 |  |  |  |
| *Least deprived* |  | — | — |  |
| *More deprived* |  | 0.70 | 0.48, 1.02 | 0.066 |
| **G1 Townsend deprivation score, quintiles (20y)** | 404 |  |  |  |
| *Least deprived* |  | — | — |  |
| *More deprived* |  | 0.64 | 0.41, 1.01 | 0.053 |
| **Is a parent (20y)** | 658 |  |  |  |
| *No* |  | — | — |  |
| *Yes* |  | 0.34 | 0.10, 0.87 | 0.045 |
| **Is a parent (22y)** | 637 |  |  |  |
| *No* |  | — | — |  |
| *Yes* |  | 0.28 | 0.10, 0.66 | 0.009 |
| *^1^*OR = Odds Ratio, CI = Confidence Interval | | | | |

Supplementary Table S5. All observed transitions between nicotine use states across 5 timepoints, from ages 21 to 30 years, where nicotine use status when missing was assumed to remain the same as that reported during the previous wave of data collection. The numbers shown in brackets refer to the first reported transition observed following smoking at age 21.

|  | | **TO** | | | | |
| --- | --- | --- | --- | --- | --- | --- |
|  |  | **Exclusive smoking** | **Dual use** | **Exclusive e-cigarette use** | **Non-use** | ***TOTAL*** |
| **FROM** | **Exclusive smoking** | - | 168 (160*) | 70 (58*) | 400 (388*) | 638 |
|  | **Dual use** | 57 | - | 25 | 21 | 103 |
|  | **Exclusive e-cigarette. use** | 6 | 14 | - | 9 | 29 |
|  | **Non-use** | 148 | 20 | 27 | - | 195 |
|  | ***TOTAL*** | 211 | 202 | 122 | 430 | 965 |

Supplementary Table S6. Average of all observed transitions between nicotine use states across 5 timepoints, from ages 21 to 30 years, where nicotine use status at all waves of data collection was imputed. The numbers shown in brackets refer to the first reported transition observed following smoking at age 21.

|  | | **TO** | | | | |
| --- | --- | --- | --- | --- | --- | --- |
|  |  | **Exclusive smoking** | **Dual use** | **Exclusive e-cig use** | **Non-use** | ***TOTAL*** |
| **FROM** | **Exclusive smoking** | - | 249 (230*) | 95 (74*) | 469 (448*) | 813 |
|  | **Dual use** | 91 | - | 51 | 36 | 178 |
|  | **Exclusive e-cigarette. use** | 17 | 29 | - | 18 | 64 |
|  | **Non-use** | 211 | 35 | 50 | - | 296 |
|  | ***TOTAL*** | 319 | 313 | 196 | 523 | 1351 |

Supplementary Table S7. Differences in characteristics between participants who did or did not report at least one transition between nicotine use states

| **Characteristic** | **0** N = 606*^1^* | **1** N = 252*^1^* | **p-value***^2^* |
| --- | --- | --- | --- |
| Sex assigned at birth |  |  | 0.015 |
| Male | 202 (33%) | 106 (42%) |  |
| Female | 404 (67%) | 146 (58%) |  |
| Ethnicity |  |  | 0.8 |
| White | 532 (97%) | 228 (96%) |  |
| BAME* | 19 (3.4%) | 9 (3.8%) |  |
| Average household income, GBP per week (11y) |  |  | 0.2 |
| 560+ | 200 (43%) | 65 (35%) |  |
| 430-559 | 99 (21%) | 42 (23%) |  |
| 240-429 | 123 (26%) | 53 (29%) |  |
| <240 | 44 (9.4%) | 25 (14%) |  |
| Parental smoking (12y) |  |  | 0.12 |
| No | 401 (77%) | 146 (72%) |  |
| Yes | 117 (23%) | 57 (28%) |  |
| Smoking frequency (21y) |  |  | <0.001 |
| Occasional | 267 (44%) | 45 (18%) |  |
| Weekly or more | 335 (56%) | 206 (82%) |  |
| Binge drinking, six or more units (20y) |  |  | 0.5 |
| Never, monthly, or less | 253 (55%) | 100 (58%) |  |
| Weekly or more | 205 (45%) | 72 (42%) |  |
| Cannabis use (20y) |  |  | <0.001 |
| Never, less than monthly, or not in past year | 378 (81%) | 111 (63%) |  |
| Monthly or more | 89 (19%) | 65 (37%) |  |
| Drug use (20y) |  |  | 0.021 |
| No | 264 (59%) | 83 (49%) |  |
| Yes | 180 (41%) | 86 (51%) |  |
| Peer smoking (20y) |  |  | 0.4 |
| None, a few, or some | 196 (42%) | 65 (38%) |  |
| Most or all | 272 (58%) | 107 (62%) |  |
| Educational qualifications (20y) |  |  | 0.035 |
| Degree-level | 108 (23%) | 26 (15%) |  |
| A-level or lower/Other | 367 (77%) | 146 (85%) |  |
| Is a parent (21y) |  |  | 0.010 |
| No | 559 (93%) | 220 (88%) |  |
| Yes | 40 (6.7%) | 30 (12%) |  |
| G1 Townsend deprivation score, quintiles (21y) |  |  | 0.081 |
| Least deprived | 218 (37%) | 75 (31%) |  |
| More deprived | 372 (63%) | 170 (69%) |  |
| BMI (18y) |  |  | >0.9 |
| <25 | 348 (78%) | 122 (78%) |  |
| >=25 | 100 (22%) | 35 (22%) |  |
| Frequency of exercise, past year (18y) |  |  | 0.060 |
| Weekly or more | 252 (67%) | 62 (57%) |  |
| Less than weekly | 123 (33%) | 46 (43%) |  |
| Mood and Feelings Questionnaire scores (21y) |  |  | 0.6 |
| <12 | 466 (79%) | 187 (78%) |  |
| >=12 | 121 (21%) | 54 (22%) |  |
| *^1^*n (%) | | | |
| *^2^*Pearson's Chi-squared test | | | |

Supplementary Table S8. Differences in characteristics by first reported transitions from smoking in those not loss to follow up, where transitions were derived by assuming transitions did not occur during any missing prior or intermediate reports of nicotine use, excluding any missingness in each characteristic

| **Characteristic** | **No nicotine use** | | | **Exclusive e-cig use** | | | **Dual use** | | |
| --- | --- | --- | --- | --- | --- | --- | --- | --- | --- |
|  | **No*^1^*** | **Yes*^1^*** | **p***^2^* | **No*^1^*** | **Yes*^1^*** | **p***^3^* | **No*^1^*** | **Yes*^1^*** | **p***^2^* |
| ***Early-life confounders*** | | | | | | | | | |
| **Sex assigned at birth** |  |  | 0.093 |  |  | 0.009 |  |  | 0.019 |
| Male | 155 (37%) | 123 (32%) |  | 267 (36%) | 11 (19%) |  | 210 (33%) | 68 (43%) |  |
| Female | 260 (63%) | 265 (68%) |  | 478 (64%) | 47 (81%) |  | 433 (67%) | 92 (58%) |  |
| **Ethnicity** | *** | | | | | | | | |
| White |  |  |  |  |  |  |  |  |  |
| Minority ethnic |  |  |  |  |  |  |  |  |  |
| **Household income (11y)** |  |  | 0.700 |  |  | 0.800 |  |  | 0.400 |
| 560+ | 124 (39%) | 129 (44%) |  | 233 (41%) | 20 (43%) |  | 202 (41%) | 51 (41%) |  |
| 430-559 | 68 (22%) | 65 (22%) |  | 121 (21%) | 12 (26%) |  | 111 (23%) | 22 (18%) |  |
| 240-429 | 86 (27%) | 74 (25%) |  | 150 (27%) | 10 (21%) |  | 121 (25%) | 39 (32%) |  |
| <240 | 36 (11%) | 28 (9.5%) |  | 59 (10%) | 5 (11%) |  | 53 (11%) | 11 (8.9%) |  |
| **Parental smoking (12y)** |  |  | <0.001 |  |  | 0.500 |  |  | 0.007 |
| No | 239 (69%) | 272 (83%) |  | 475 (76%) | 36 (72%) |  | 418 (78%) | 93 (67%) |  |
| Yes | 107 (31%) | 57 (17%) |  | 150 (24%) | 14 (28%) |  | 118 (22%) | 46 (33%) |  |
| ***Substance use*** | | | | | | | | | |
| **Smoking frequency (21y)** |  |  | <0.001 |  |  | 0.003 |  |  | <0.001 |
| Occasional | 70 (17%) | 225 (58%) |  | 285 (38%) | 10 (18%) |  | 263 (41%) | 32 (20%) |  |
| Weekly or more | 341 (83%) | 162 (42%) |  | 458 (62%) | 45 (82%) |  | 375 (59%) | 128 (80%) |  |
| **Binge drinking frequency (20y)** |  |  | 0.600 |  |  | 0.800 |  |  | >0.9 |
| Never, monthly, or less | 173 (57%) | 164 (55%) |  | 314 (56%) | 23 (55%) |  | 271 (56%) | 66 (56%) |  |
| Weekly or more | 129 (43%) | 134 (45%) |  | 244 (44%) | 19 (45%) |  | 211 (44%) | 52 (44%) |  |
| **Cannabis use (20y)** |  |  | <0.001 |  |  | 0.800 |  |  | 0.700 |
| Never, less than monthly, or not in past year | 216 (70%) | 252 (83%) |  | 436 (76%) | 32 (78%) |  | 374 (76%) | 94 (78%) |  |
| Monthly or more | 92 (30%) | 53 (17%) |  | 136 (24%) | 9 (22%) |  | 118 (24%) | 27 (22%) |  |
| **Drug use (20y)** |  |  | 0.009 |  |  | 0.200 |  |  | 0.800 |
| No | 151 (51%) | 180 (62%) |  | 312 (57%) | 19 (46%) |  | 266 (56%) | 65 (58%) |  |
| Yes | 143 (49%) | 110 (38%) |  | 231 (43%) | 22 (54%) |  | 205 (44%) | 48 (42%) |  |
| ***Social and sociodemographic factors*** | | | | | | | | | |
| **Peer smoking (20y)** |  |  | <0.001 |  |  | 0.009 |  |  | 0.034 |
| None, a few, or some | 98 (32%) | 149 (49%) |  | 238 (42%) | 9 (21%) |  | 209 (43%) | 38 (32%) |  |
| Most or all | 205 (68%) | 158 (51%) |  | 330 (58%) | 33 (79%) |  | 282 (57%) | 81 (68%) |  |
| **Education (20y)** |  |  | <0.001 |  |  | 0.500 |  |  | 0.044 |
| Degree-level | 46 (15%) | 81 (26%) |  | 117 (20%) | 10 (24%) |  | 110 (22%) | 17 (14%) |  |
| A-level or lower/Other | 260 (85%) | 231 (74%) |  | 460 (80%) | 31 (76%) |  | 386 (78%) | 105 (86%) |  |
| **Being a parent (21y)** |  |  | 0.006 |  |  | 0.800 |  |  | 0.400 |
| No | 367 (90%) | 365 (95%) |  | 680 (92%) | 52 (91%) |  | 590 (92%) | 142 (90%) |  |
| Yes | 43 (10%) | 20 (5.2%) |  | 58 (7.9%) | 5 (8.8%) |  | 48 (7.5%) | 15 (9.6%) |  |
| **Neighbourhood deprivation (21y)** |  |  | 0.200 |  |  | 0.800 |  |  | 0.700 |
| Least deprived quintile | 133 (33%) | 143 (38%) |  | 258 (35%) | 18 (34%) |  | 219 (35%) | 57 (36%) |  |
| More deprived quintiles | 271 (67%) | 237 (62%) |  | 473 (65%) | 35 (66%) |  | 408 (65%) | 100 (64%) |  |
| ***Physical and mental health*** | | | | | | | | | |
| **BMI*^4^* (18y)** |  |  | 0.200 |  |  | 0.200 |  |  | 0.018 |
| <25 | 219 (75%) | 232 (80%) |  | 417 (77%) | 34 (85%) |  | 369 (80%) | 82 (69%) |  |
| >=25 | 72 (25%) | 58 (20%) |  | 124 (23%) | 6 (15%) |  | 94 (20%) | 36 (31%) |  |
| **Exercise frequency (18y)** |  |  | 0.010 |  |  | 0.120 |  |  | 0.800 |
| Weekly or more | 128 (59%) | 176 (70%) |  | 286 (66%) | 18 (53%) |  | 246 (65%) | 58 (64%) |  |
| Less than weekly | 89 (41%) | 74 (30%) |  | 147 (34%) | 16 (47%) |  | 130 (35%) | 33 (36%) |  |
| **Depressive symptoms (21y)** |  |  | 0.200 |  |  | 0.500 |  |  | 0.300 |
| <12 SFMQ*^5^* score | 312 (77%) | 302 (81%) |  | 570 (79%) | 44 (76%) |  | 494 (80%) | 120 (76%) |  |
| >=12 SFMQ score | 91 (23%) | 70 (19%) |  | 147 (21%) | 14 (24%) |  | 124 (20%) | 37 (24%) |  |
| *^1^* n (%);*^2^* Pearson's Chi-squared test; *^3^* Pearson's Chi-squared test; Fisher's exact test; *^4^* BMI = Body Mass Index; *^5^* SMFQ = Short Mood and Feelings Questionnaire; *** Omitted to avoid identification of small cell counts (<5) due to the low number of participants from a minority ethnic background | | | | | | | | | |

Supplementary Table S9. Differences in characteristics by early-life confounders excluding any pairwise missingness

|  | **Sex assigned at birth** | | | **Household income** | | | | | **Parental smoking** | | |
| --- | --- | --- | --- | --- | --- | --- | --- | --- | --- | --- | --- |
| **Characteristic** | **Male***^1^* | **Female***^1^* | **p***^2^* | **560+***^1^* | **430-559***^1^* | **240-429***^1^* | **<240***^1^* | **p***^3^* | **No***^1^* | **Yes***^1^* | **p***^2^* |
| ***Early-life confounders*** | | | | | | | | | | | |
| **Sex assigned at birth** |  |  |  |  |  |  |  | 0.140 |  |  | 0.900 |
| Male |  |  |  | 118 (45%) | 53 (38%) | 64 (36%) | 22 (32%) |  | 210 (38%) | 68 (39%) |  |
| Female |  |  |  | 147 (55%) | 88 (62%) | 112 (64%) | 47 (68%) |  | 337 (62%) | 106 (61%) |  |
| **Ethnicity** | *** | | | | | | | | | | |
| White |  |  |  |  |  |  |  |  |  |  |  |
| Minority ethnic |  |  |  |  |  |  |  |  |  |  |  |
| **Household income (11y)** |  |  | 0.140 |  |  |  |  |  |  |  | 0.009 |
| 560+ | 118 (46%) | 147 (37%) |  |  |  |  |  |  | 211 (45%) | 42 (31%) |  |
| 430-559 | 53 (21%) | 88 (22%) |  |  |  |  |  |  | 100 (21%) | 31 (23%) |  |
| 240-429 | 64 (25%) | 112 (28%) |  |  |  |  |  |  | 120 (26%) | 44 (32%) |  |
| <240 | 22 (8.6%) | 47 (12%) |  |  |  |  |  |  | 38 (8.1%) | 20 (15%) |  |
| **Parental smoking (12y)** |  |  | 0.900 |  |  |  |  | 0.009 |  |  |  |
| No | 210 (76%) | 337 (76%) |  | 211 (83%) | 100 (76%) | 120 (73%) | 38 (66%) |  |  |  |  |
| Yes | 68 (24%) | 106 (24%) |  | 42 (17%) | 31 (24%) | 44 (27%) | 20 (34%) |  |  |  |  |
| ***Substance use*** | | | | | | | | | | | |
| **Smoking frequency (21y)** |  |  | 0.300 |  |  |  |  | 0.079 |  |  | <0.001 |
| Occasional | 120 (39%) | 192 (35%) |  | 111 (42%) | 51 (36%) | 57 (33%) | 19 (28%) |  | 236 (43%) | 36 (21%) |  |
| Weekly or more | 187 (61%) | 354 (65%) |  | 153 (58%) | 89 (64%) | 116 (67%) | 50 (72%) |  | 307 (57%) | 137 (79%) |  |
| **Binge drinking frequency (20y)** |  |  | <0.001 |  |  |  |  | 0.016 |  |  | 0.081 |
| Never, monthly, or less | 100 (44%) | 253 (63%) |  | 99 (47%) | 53 (52%) | 72 (63%) | 33 (65%) |  | 216 (53%) | 78 (62%) |  |
| Weekly or more | 126 (56%) | 151 (37%) |  | 111 (53%) | 48 (48%) | 42 (37%) | 18 (35%) |  | 191 (47%) | 48 (38%) |  |
| **Cannabis use (20y)** |  |  | <0.001 |  |  |  |  | 0.093 |  |  | 0.150 |
| Never, less than monthly, or not past year | 150 (66%) | 339 (82%) |  | 148 (71%) | 84 (81%) | 96 (79%) | 34 (67%) |  | 321 (78%) | 91 (72%) |  |
| Monthly or more | 78 (34%) | 76 (18%) |  | 61 (29%) | 20 (19%) | 26 (21%) | 17 (33%) |  | 91 (22%) | 36 (28%) |  |
| **Drug use (20y)** |  |  | <0.001 |  |  |  |  | 0.700 |  |  | 0.700 |
| No | 100 (46%) | 247 (62%) |  | 108 (53%) | 55 (55%) | 67 (60%) | 28 (57%) |  | 223 (57%) | 66 (55%) |  |
| Yes | 116 (54%) | 150 (38%) |  | 97 (47%) | 45 (45%) | 45 (40%) | 21 (43%) |  | 170 (43%) | 55 (45%) |  |
| ***Social and sociodemographic factors*** | | | | | | | | | | | |
| **Peer smoking (20y)** |  |  | 0.200 |  |  |  |  | >0.9 |  |  | 0.100 |
| None, a few, or some | 99 (44%) | 162 (39%) |  | 87 (41%) | 42 (41%) | 48 (39%) | 19 (37%) |  | 180 (44%) | 45 (35%) |  |
| Most or all | 126 (56%) | 253 (61%) |  | 123 (59%) | 60 (59%) | 74 (61%) | 32 (63%) |  | 232 (56%) | 82 (65%) |  |
| **Education (20y)** |  |  | 0.900 |  |  |  |  | 0.5 |  |  | 0.200 |
| Degree-level | 47 (20%) | 87 (21%) |  | 50 (23%) | 28 (27%) | 26 (22%) | 8 (15%) |  | 99 (24%) | 23 (18%) |  |
| A-level or lower/Other | 184 (80%) | 329 (79%) |  | 165 (77%) | 77 (73%) | 93 (78%) | 44 (85%) |  | 317 (76%) | 103 (82%) |  |
| **Being a parent (21y)** |  |  | <0.001 |  |  |  |  | <0.001 |  |  | 0.6 |
| No | 293 (96%) | 486 (89%) |  | 255 (97%) | 132 (95%) | 154 (88%) | 62 (91%) |  | 499 (92%) | 163 (94%) |  |
| Yes | 11 (3.6%) | 59 (11%) |  | 7 (2.7%) | 7 (5.0%) | 21 (12%) | 6 (8.8%) |  | 41 (7.6%) | 11 (6.3%) |  |
| **Neighbourhood deprivation (21y)** |  |  | 0.120 |  |  |  |  | <0.001 |  |  | 0.020 |
| Least deprived quintile | 114 (39%) | 179 (33%) |  | 114 (44%) | 49 (36%) | 48 (28%) | 14 (21%) |  | 205 (38%) | 48 (29%) |  |
| More deprived quintiles | 182 (61%) | 360 (67%) |  | 144 (56%) | 87 (64%) | 124 (72%) | 54 (79%) |  | 328 (62%) | 120 (71%) |  |
| ***Physical and mental health*** | | | | | | | | | | | |
| **BMI*^4^* (18y)** |  |  | 0.041 |  |  |  |  | 0.006 |  |  | 0.400 |
| <25 | 192 (82%) | 278 (75%) |  | 174 (86%) | 85 (77%) | 91 (71%) | 27 (69%) |  | 331 (80%) | 91 (76%) |  |
| >=25 | 42 (18%) | 93 (25%) |  | 29 (14%) | 26 (23%) | 37 (29%) | 12 (31%) |  | 84 (20%) | 29 (24%) |  |
| **Exercise frequency (18y)** |  |  | <0.001 |  |  |  |  | 0.047 |  |  | 0.083 |
| Weekly or more | 124 (76%) | 190 (60%) |  | 120 (74%) | 44 (62%) | 61 (59%) | 25 (63%) |  | 227 (68%) | 52 (58%) |  |
| Less than weekly | 40 (24%) | 129 (40%) |  | 42 (26%) | 27 (38%) | 43 (41%) | 15 (38%) |  | 109 (32%) | 38 (42%) |  |
| **Depressive symptoms (21y)** |  |  | <0.001 |  |  |  |  | >0.9 |  |  | 0.500 |
| <12 SFMQ*^5^* score | 256 (86%) | 397 (75%) |  | 205 (80%) | 106 (79%) | 136 (79%) | 53 (79%) |  | 420 (80%) | 139 (82%) |  |
| >=12 SFMQ score | 40 (14%) | 135 (25%) |  | 52 (20%) | 29 (21%) | 37 (21%) | 14 (21%) |  | 107 (20%) | 30 (18%) |  |
| *^1^* n (%); *^2^* Pearson's Chi-squared test; *^3^* Pearson's Chi-squared test; Fisher's exact test; *^4^* BMI = Body Mass Index; *^5^* SMFQ = Short Mood and Feelings Questionnaire; *** Omitted to avoid identification of small cell counts (<5) due to the low number of participants from a minority ethnic background | | | | | | | | | | | |

Supplementary Table S10. Pooled summary statistics for *early-life confounders* and time from sub-distribution discrete time survival analyses, weighted for selection via smoking

|  | **No nicotine use (52%)** | | | **Exclusive e-cig use (9%)** | | | **Dual use (27%)** | | |
| --- | --- | --- | --- | --- | --- | --- | --- | --- | --- |
| **Characteristic** | **SHR***^3^* | **95% CI***^1^* | **p** | **SHR***^3^* | **95% CI***^1^* | **p** | **SHR***^3^* | **95% CI***^1^* | **p** |
| **Years since 21+ questionnaire*^4^*** | 0.72 | 0.65, 0.79 | <0.001 | 1.05 | 0.82, 1.36 | 0.7 | 0.87 | 0.76, 0.99 | 0.04 |
| **Sex assigned at birth** |  |  |  |  |  |  |  |  |  |
| *Male* | — | — |  | — | — |  | — | — |  |
| *Female* | 1.25 | 0.98, 1.59 | 0.07 | 1.62 | 0.75, 3.49 | 0.2 | 0.69 | 0.49, 0.98 | 0.036 |
| **Ethnicity** |  |  |  |  |  |  |  |  |  |
| *White* | — | — |  | — | — |  | — | — |  |
| *Minority ethnic* | 1.3 | 0.72, 2.37 | 0.4 | 0.01 | 0.00, 4,582 | 0.5 | 0.71 | 0.25, 2.05 | 0.5 |
| **Household income (11y)** |  |  |  |  |  |  |  |  |  |
| *560+* | — | — |  | — | — |  | — | — |  |
| *430-559* | 0.98 | 0.71, 1.35 | >0.9 | 0.97 | 0.42, 2.23 | >0.9 | 0.8 | 0.49, 1.31 | 0.4 |
| *240-429* | 0.95 | 0.71, 1.27 | 0.7 | 0.96 | 0.42, 2.17 | >0.9 | 1.19 | 0.78, 1.80 | 0.4 |
| *<240* | 0.8 | 0.53, 1.20 | 0.3 | 0.89 | 0.28, 2.83 | 0.8 | 0.93 | 0.49, 1.77 | 0.8 |
| **Parental smoking (12y)** |  |  |  |  |  |  |  |  |  |
| *No* | — | — |  | — | — |  | — | — |  |
| *Yes* | 0.6 | 0.44, 0.81 | 0.001 | 1.27 | 0.63, 2.57 | 0.5 | 1.58 | 1.08, 2.32 | 0.02 |
| *^1^* SHR = Sub-distribution Hazard Ratio; *^2^* Discrete-time indicator where coefficients represent the effect of a one-unit increase in the time interval on the sub-distribution hazard ratio | | | | | | | | | |

Supplementary Table S11. Pooled summary statistics from sub-distribution discrete time survival analyses, adjusted for early-life confounders, *unweighted*

| **Characteristic** | **No nicotine use (52%)** | | | **Exclusive e-cig use (9%)** | | | **Dual use (27%)** | | |
| --- | --- | --- | --- | --- | --- | --- | --- | --- | --- |
|  | **SHR***^1^* | **95% CI***^1^* | **p** | **SHR***^1^* | **95% CI***^1^* | **p** | **SHR***^1^* | **95% CI***^1^* | **p** |
| ***Substance use*** | | | | | | | | | |
| **Smoking frequency (21y)** |  |  |  |  |  |  |  |  |  |
| Occasional | — | — |  | — | — |  | — | — |  |
| Weekly or more | 0.28 | 0.22, 0.34 | <0.001 | 2.64 | 1.27, 5.47 | 0.009 | 2.60 | 1.75, 3.87 | <0.001 |
| **Binge drinking frequency (20y)** |  |  |  |  |  |  |  |  |  |
| Never, monthly, or less | — | — |  | — | — |  | — | — |  |
| Weekly or more | 1.03 | 0.81, 1.30 | 0.8 | 1.16 | 0.62, 2.18 | 0.6 | 0.92 | 0.65, 1.30 | 0.6 |
| **Cannabis use (20y)** |  |  |  |  |  |  |  |  |  |
| Never, less than monthly, not past year | — | — |  | — | — |  | — | — |  |
| Monthly or more | 0.65 | 0.48, 0.87 | 0.004 | 1.01 | 0.48, 2.14 | >0.9 | 1.15 | 0.79, 1.66 | 0.5 |
| **Drug use (20y)** |  |  |  |  |  |  |  |  |  |
| No | — | — |  | — | — |  | — | — |  |
| Yes | 0.75 | 0.59, 0.95 | 0.015 | 1.36 | 0.75, 2.46 | 0.3 | 1.03 | 0.74, 1.45 | 0.8 |
| ***Social and sociodemographic factors*** | | | | | | | | | |
| **Peer smoking (20y)** |  |  |  |  |  |  |  |  |  |
| None, a few, or some | — | — |  | — | — |  | — | — |  |
| Most or all | 0.62 | 0.50, 0.78 | <0.001 | 1.82 | 0.89, 3.74 | 0.10 | 1.43 | 1.01, 2.04 | 0.046 |
| **Education (20y)** |  |  |  |  |  |  |  |  |  |
| Degree-level | — | — |  | — | — |  | — | — |  |
| A-level or lower/Other | 0.68 | 0.52, 0.88 | 0.004 | 0.72 | 0.36, 1.42 | 0.3 | 1.64 | 1.02, 2.66 | 0.043 |
| **Being a parent (21y)** |  |  |  |  |  |  |  |  |  |
| No | — | — |  | — | — |  | — | — |  |
| Yes | 0.51 | 0.32, 0.83 | 0.006 | 1.08 | 0.37, 3.13 | 0.9 | 1.28 | 0.76, 2.15 | 0.3 |
| **Neighbourhood deprivation (21y)** |  |  |  |  |  |  |  |  |  |
| Least deprived quintile | — | — |  | — | — |  | — | — |  |
| More deprived quintiles | 0.91 | 0.73, 1.14 | 0.4 | 0.95 | 0.52, 1.75 | 0.9 | 0.97 | 0.70, 1.33 | 0.8 |
| ***Physical and mental health*** | | | | | | | | | |
| **BMI*^2^* (18y)** |  |  |  |  |  |  |  |  |  |
| <25 | — | — |  | — | — |  | — | — |  |
| >=25 | 0.91 | 0.68, 1.20 | 0.5 | 0.63 | 0.26, 1.48 | 0.3 | 1.59 | 1.11, 2.27 | 0.011 |
| **Exercise frequency (18y)** |  |  |  |  |  |  |  |  |  |
| Weekly or more | — | — |  | — | — |  | — | — |  |
| Less than weekly | 0.71 | 0.54, 0.94 | 0.018 | 1.20 | 0.60, 2.40 | 0.6 | 1.10 | 0.75, 1.61 | 0.6 |
| **Depressive symptoms (21y)** |  |  |  |  |  |  |  |  |  |
| <12 SMFQ*^3^* score | — | — |  | — | — |  | — | — |  |
| >=12 SMFQ score | 0.79 | 0.61, 1.03 | 0.079 | 1.12 | 0.59, 2.11 | 0.7 | 1.14 | 0.81, 1.63 | 0.4 |
| *^1^* SHR = Sub-distribution Hazard Ratio, CI = Confidence Interval; *^2^* BMI = Body Mass Index; *^3^* SMFQ = Short Mood and Feelings Questionnaire | | | | | | | | | |

Supplementary Table S12. Pooled summary statistics from sub-distribution discrete time survival analyses, *unadjusted*, weighted for selection via smoking

| **Characteristic** | **No nicotine use (52%)** | | | **Exclusive e-cig use (9%)** | | | **Dual use (27%)** | | |
| --- | --- | --- | --- | --- | --- | --- | --- | --- | --- |
|  | **SHR***^1^* | **95% CI***^1^* | **p** | **SHR***^1^* | **95% CI***^1^* | **p** | **SHR***^1^* | **95% CI***^1^* | **p** |
| ***Substance use*** | | | | | | | | | |
| **Smoking frequency (21y)** |  |  |  |  |  |  |  |  |  |
| Occasional | — | — |  | — | — |  | — | — |  |
| Weekly or more | 0.28 | 0.22, 0.35 | <0.001 | 2.92 | 1.31, 6.49 | 0.009 | 3.03 | 1.98, 4.63 | <0.001 |
| **Binge drinking (20y)** |  |  |  |  |  |  |  |  |  |
| Never, monthly, or less | — | — |  | — | — |  | — | — |  |
| Weekly or more | 1.01 | 0.79, 1.29 | >0.9 | 1.10 | 0.56, 2.16 | 0.8 | 0.95 | 0.66, 1.37 | 0.8 |
| **Cannabis use (20y)** |  |  |  |  |  |  |  |  |  |
| Never, less than monthly, not past year | — | — |  | — | — |  | — | — |  |
| Monthly or more | 0.64 | 0.47, 0.87 | 0.005 | 0.98 | 0.45, 2.14 | >0.9 | 1.27 | 0.85, 1.90 | 0.3 |
| **Drug use (20y)** |  |  |  |  |  |  |  |  |  |
| No | — | — |  | — | — |  | — | — |  |
| Yes | 0.76 | 0.60, 0.98 | 0.032 | 1.35 | 0.71, 2.58 | 0.4 | 1.11 | 0.78, 1.60 | 0.6 |
| ***Social and sociodemographic factors*** | | | | | | | | | |
| **Peer smoking (20y)** |  |  |  |  |  |  |  |  |  |
| None, a few, or some | — | — |  | — | — |  | — | — |  |
| Most or all | 0.64 | 0.51, 0.81 | <0.001 | 1.76 | 0.83, 3.70 | 0.14 | 1.53 | 1.06, 2.19 | 0.022 |
| **Education (20y)** |  |  |  |  |  |  |  |  |  |
| Degree-level | — | — |  | — | — |  | — | — |  |
| A-level or lower/Other | 0.68 | 0.51, 0.89 | 0.005 | 0.66 | 0.32, 1.37 | 0.3 | 1.77 | 1.05, 2.96 | 0.031 |
| **Being a parent (21y)** |  |  |  |  |  |  |  |  |  |
| No | — | — |  | — | — |  | — | — |  |
| Yes | 0.49 | 0.29, 0.82 | 0.007 | 1.37 | 0.44, 4.27 | 0.6 | 1.46 | 0.86, 2.50 | 0.2 |
| **Neighbourhood deprivation (21y)** |  |  |  |  |  |  |  |  |  |
| Least deprived quintile | — | — |  | — | — |  | — | — |  |
| More deprived quintiles | 0.90 | 0.72, 1.13 | 0.4 | 1.06 | 0.54, 2.07 | 0.9 | 1.01 | 0.72, 1.43 | >0.9 |
| ***Physical and mental health*** | | | | | | | | | |
| **BMI*^2^* (18y)** |  |  |  |  |  |  |  |  |  |
| <25 | — | — |  | — | — |  | — | — |  |
| >=25 | 0.88 | 0.66, 1.16 | 0.3 | 0.67 | 0.27, 1.64 | 0.4 | 1.56 | 1.07, 2.26 | 0.019 |
| **Frequency of exercise, past year (18y)** |  |  |  |  |  |  |  |  |  |
| Weekly or more | — | — |  | — | — |  | — | — |  |
| Less than weekly | 0.73 | 0.55, 0.97 | 0.030 | 1.40 | 0.68, 2.89 | 0.4 | 1.03 | 0.68, 1.55 | 0.9 |
| **Depressive symptoms (21y)** |  |  |  |  |  |  |  |  |  |
| <12 SMFQ*^3^* score | — | — |  | — | — |  | — | — |  |
| >=12 SMFQ score | 0.89 | 0.68, 1.17 | 0.4 | 1.20 | 0.61, 2.37 | 0.6 | 1.15 | 0.79, 1.68 | 0.5 |
| *^1^* SHR = Sub-distribution Hazard Ratio, CI = Confidence Interval; *^2^* BMI = Body Mass Index; *^3^* SMFQ = Short Mood and Feelings Questionnaire | | | | | | | | | |

Supplementary Table S13. Summary statistics from sub-distribution discrete time survival, adjusted for early-life confounders, *complete cases* (n=168), unweighted (as weights rely on imputed data)

| **Characteristic** | **No nicotine use (n=90)** | | | **Exclusive e-cig use (n=12)** | | | **Dual use (n=36)** | | |
| --- | --- | --- | --- | --- | --- | --- | --- | --- | --- |
|  | **SHR***^1^* | **95% CI***^1^* | **p** | **SHR***^1^* | **95% CI***^1^* | **p** | **SHR***^1^* | **95% CI***^1^* | **p** |
| ***Substance use*** | | | | | | | | | |
| **Smoking frequency (21y)** |  |  |  |  |  |  |  |  |  |
| Occasional | — | — |  | — | — |  | — | — |  |
| Weekly or more | 0.25 | 0.16, 0.40 | <0.001 | 3.24 | 0.93, 14.9 | 0.085 | 2.27 | 1.12, 4.90 | 0.028 |
| **Binge drinking (20y)** |  |  |  |  |  |  |  |  |  |
| Never, monthly, or less | — | — |  | — | — |  | — | — |  |
| Weekly or more | 0.96 | 0.62, 1.49 | 0.9 | 2.10 | 0.63, 8.17 | 0.2 | 0.83 | 0.41, 1.65 | 0.6 |
| **Cannabis use (20y)** |  |  |  |  |  |  |  |  |  |
| Never, less than monthly, not past year | — | — |  | — | — |  | — | — |  |
| Monthly or more | 0.46 | 0.24, 0.83 | 0.014 | 1.45 | 0.37, 4.93 | 0.6 | 1.24 | 0.55, 2.57 | 0.6 |
| **Drug use (20y)** |  |  |  |  |  |  |  |  |  |
| No | — | — |  | — | — |  | — | — |  |
| Yes | 0.59 | 0.37, 0.93 | 0.023 | 1.77 | 0.55, 6.22 | 0.3 | 1.19 | 0.60, 2.34 | 0.6 |
| ***Social and sociodemographic factors*** | | | | | | | | | |
| **Peer smoking (20y)** |  |  |  |  |  |  |  |  |  |
| None, a few, or some | — | — |  | — | — |  | — | — |  |
| Most or all | 0.60 | 0.39, 0.94 | 0.022 | 1.18 | 0.36, 4.54 | 0.8 | 1.57 | 0.77, 3.38 | 0.2 |
| **Education (20y)** |  |  |  |  |  |  |  |  |  |
| Degree-level | — | — |  | — | — |  | — | — |  |
| A-level or lower/Other | 0.42 | 0.27, 0.68 | <0.001 | 1.63 | 0.41, 10.9 | 0.5 | 3.61 | 1.28, 15.1 | 0.035 |
| **Being a parent (21y)** |  |  |  |  |  |  |  |  |  |
| No | — | — |  | — | — |  | — | — |  |
| Yes | 0.00 |  | >0.9 | 0.00 |  | >0.9 | 4.98 | 1.05, 18.3 | 0.023 |
| **Neighbourhood deprivation (21y)** |  |  |  |  |  |  |  |  |  |
| Least deprived quintile | — | — |  | — | — |  | — | — |  |
| More deprived quintiles | 1.01 | 0.65, 1.57 | >0.9 | 0.92 | 0.27, 3.18 | 0.9 | 0.77 | 0.39, 1.54 | 0.5 |
| ***Physical and mental health*** | | | | | | | | | |
| **BMI^2^ (18y)** |  |  |  |  |  |  |  |  |  |
| <25 | — | — |  | — | — |  | — | — |  |
| >=25 | 0.87 | 0.46, 1.54 | 0.6 | 0.40 | 0.02, 2.15 | 0.4 | 1.45 | 0.60, 3.13 | 0.4 |
| **Exercise frequency (18y)** |  |  |  |  |  |  |  |  |  |
| Weekly or more | — | — |  | — | — |  | — | — |  |
| Less than weekly | 0.59 | 0.36, 0.93 | 0.028 | 1.62 | 0.47, 5.30 | 0.4 | 1.36 | 0.67, 2.68 | 0.4 |
| **Depressive symptoms (21y)** |  |  |  |  |  |  |  |  |  |
| <12 SMFQ^3^ score | — | — |  | — | — |  | — | — |  |
| >=12 SMFQ score | 0.78 | 0.41, 1.37 | 0.4 | 2.12 | 0.46, 7.48 | 0.3 | 1.11 | 0.41, 2.54 | 0.8 |
| *^1^* SHR = Sub-distribution Hazard Ratio, CI = Confidence Interval; *^2^* BMI = Body Mass Index; *^3^* SMFQ = Short Mood and Feelings Questionnaire | | | | | | | | | |
